# *Terfezia boudieri* and *Terfezia claveryi* inhibit the LPS/IFN-γ-mediated inflammation in RAW 264.7 macrophages through an Nrf2-independent mechanism

**DOI:** 10.1038/s41598-023-35612-8

**Published:** 2023-06-21

**Authors:** Abdelhameed S. Dawood, Mohamed S. Sedeek, Mohamed A. Farag, Anwar Abdelnaser

**Affiliations:** 1 Biotechnology Graduate Program, School of Sciences and Engineering, The American University in Cairo, New Cairo, 11835 Egypt; 2 Institute of Global Health and Human Ecology, School of Sciences and Engineering, The American University in Cairo, P.O. Box: 74, New Cairo, 11835 Egypt; 3 Pharmacognosy Department, College of Pharmacy, Cairo University, Kasr El Aini St., Cairo, 11562 Egypt

**Keywords:** Innate immune cells, Genetics, Cancer, Infectious diseases, Immunochemistry, Metabolomics

## Abstract

Desert truffles have been used as traditional treatments for numerous inflammatory disorders. However, the molecular mechanisms underlying their anti-inflammatory effects in RAW 264.7 macrophages have yet to be fully elucidated. The present study investigated the anti-inflammatory activities of two main desert truffles, *Terfezia boudieri* and *T. claveryi,* and the underlying mechanisms associated with their anti-inflammatory activities in RAW 264.7 macrophages stimulated with lipopolysaccharide/interferon-gamma (LPS/IFN-γ). Our results demonstrated that treatment with *T. boudieri* and *T. claveryi* extracts effectively suppressed the inflammatory response in LPS/IFN-γ-stimulated RAW 264.7 macrophages. Specifically, *T. boudieri* extract was found to reduce the production of nitric oxide and inhibit the expression of various pro-inflammatory markers, including inducible nitric oxide synthase, cyclooxygenase-2 (COX-2), tumor necrosis factor-α, and interleukin-6 (IL-6) at both the mRNA and protein levels. Similarly, *T. claveryi* extract exhibited comparable inhibitory effects, except for the expression of IL-6 and COX-2 at the protein level, where no significant effect was observed. Moreover, both studied extracts significantly downregulated the microRNA expression levels of miR-21, miR-146a, and miR-155, suggesting that *T. boudieri* and *T. claveryi* suppress the inflammatory response in LPS/IFN-γ-stimulated RAW 264.7 cells through an epigenetic mechanism. Furthermore, our study reveals a new mechanism for the anti-inflammatory properties of desert truffle extracts. We show for the first time that *Terfezia* extracts do not rely on the nuclear factor erythroid 2-related factor 2 pathway, previously linked to anti-inflammatory responses. This expands our understanding of natural product anti-inflammatory mechanisms and could have important implications for developing new therapies. To account for differences in truffle effects, extracts prepared were subjected to secondary metabolites profiling using UPLC-MS. UPLC-MS led to the annotation of 87 secondary metabolites belonging to various classes, including amino acids, carbohydrates, alkaloids, amides, fatty acids, sterols, and phenolic compounds. Therefore, these results indicate that *T. boudieri* and *T. claveryi* exhibit anti-inflammatory activities through suppressing multiple inflammatory mediators and cytokines and may be potential anti-inflammatory agents.

## Introduction

Inflammation is a protective immune response that is crucial in eliminating pathogens and maintaining the tissue homeostasis^1,2^. However, the uncontrolled or persistent inflammatory response can contribute to the pathogenesis of several chronic inflammatory diseases, including arthritis, asthma, autoimmune diseases, atherosclerosis, cancer, and diabetes^3^. Inflammation is induced when innate immune cells, particularly macrophages, detect harmful stimuli like infection and tissue injury by activating germline-encoded pattern-recognition receptors (PRRs)^4,5^. These receptors can recognize pathogen-associated molecular patterns (PAMPs), such as lipopolysaccharides (LPS), a component of the gram-negative bacterial cell wall, or damage-associated molecular patterns (DAMPs) produced from injured cells^6,7^. Notably, LPS can trigger inflammation-associated signaling pathways in macrophages through binding to the Toll-like receptors 4 (TLR4), which are then dimerized on the cell membrane. Receptor dimerization leads to the recruitment of myeloid differentiation primary response 88 (MyD88) and Toll/interleukin-1 receptor domain-containing adapter inducing interferon-β (TRIF) adaptor proteins to the intracellular domain of TLR4. Signaling via TLR4 activates an intracellular signaling cascade that results in activation of nuclear factor-kappa B (NF-κB), and mitogen-activated protein kinases (MAPKs), which eventually promote overproduction of the inflammatory mediators and cytokines^8–11^. The representative inflammatory mediators include nitric oxide (NO), histamine, prostaglandin E2 (PGE2), and pro-inflammatory cytokines, such as tumor necrosis factor-α (TNF-α), and interleukin-6 (IL-6)^12–14^. Furthermore, inflammation is known to elevate the level of intracellular reactive oxygen species (ROS), resulting in oxidative stress^15,16^. In this context, the transcription factor nuclear factor-erythroid 2-related factor 2 (Nrf2) has a pivotal role in protecting cells against inflammation and oxidative stress through modulating the expression of phase II detoxifying enzymes, such as heme oxygenase-1 (HO-1) and oxidative stress response genes, such as oxidative stress-induced growth inhibitor 1 (OSGIN1)^17–19^.

Currently, nonsteroidal anti-inflammatory drugs (NSAIDs) are the most widely prescribed drugs to treat both acute and chronic inflammatory disorders. However, their long-term use is associated with serious adverse effects, such as peptic ulcers, cardiovascular toxicity, hypertension, acute renal failure, and nephrotic syndrome^20,21^. Therefore, there is an imperative need for research on developing new natural-based anti-inflammatory compounds without side effects as alternatives to the current NSAIDs^22,23^. In this regard, truffles have a long history of use as traditional remedies to treat many human diseases, especially eye and skin diseases^24^.

Truffles are the hypogeous fruiting bodies of the ascomycetes fungi that thrive underground in depth between 5 and 10 centimeters^25^. Truffles have traditionally been utilized as functional foods and therapeutic agents due to their high content of amino acids, proteins, carbohydrates, fats, lipids, and minerals^26,27^. Moreover, they contain abundant bioactive compounds, such as phenolics, phytosterols, polysaccharides, flavonoids, and terpenoids, which are associated with their anti-inflammatory, immunomodulatory, antioxidant, anticancer, and antibacterial properties^28–30^. Edible truffles are taxonomically classified into two major types, tuber, and desert. Truffle species within the genus *Tuber* are recognized as "true" truffles, such as *Tuber magnatum* (white truffle) and *Tuber melanosporum* (black truffle), growing natively in Europe^24^. On the other hand, desert truffle species existing in arid and semi-arid areas of the Mediterranean belong to the genera *Terfezia* and *Tirmania*^28^. The genus *Terfezia* is composed of more than 20 species. *T. boudieri* and *T. claveryi* are among most popular desert truffle species^31^.

In the present study, the anti-inflammatory activities of ethanolic extracts from *T. boudieri* and *T. claveryi* have been investigated. Both species have been selected for the study based on the abundance of functional anti-inflammatory compounds in their chemical profile. Therefore, the aim of this study was to examine and compare the anti-inflammatory properties of *T. boudieri* and *T. claveryi* extracts in LPS/IFN-γ-stimulated RAW 264.7 macrophages, as well as to identify the underlying mechanisms related to these properties. Further, metabolites profiling using ultra-performance liquid chromatography-mass spectrometry was employed to provide a holistic overview of truffles metabolome.

## Materials and methods

### Materials

Dulbecco's Modified Eagle Medium (DMEM), high glucose (Cat. No. 41965-039), Fetal Bovine Serum (FBS; Cat. No. 10270-106), Penicillin-Streptomycin (Pen/Strep; Cat. No. 15140122), (3-(4, 5-dimethylthiazol-2-yl)-2, 5-diphenyltetrazolium bromide (MTT; Cat. No. M6494), Griess Reagent Kit (Cat. No. G7921), Isopropanol (HPLC grade; Cat. No. 67-63-0), Ethanol (HPLC grade; Cat. No. 64-17-5), RevertAid First Strand cDNA Synthesis Kit (Cat. No. K16.22), PowerUp™ SYBR™ Green Master Mix (Cat. No. A25741), mRNA primers (iNOS, TNF-α, IL-6, COX-2 and GAPDH), Pierce™ BCA Protein Assay Kit (Cat. No. 23225), NuPAGE™ LDS Sample Buffer (4X; Cat. No. NP0007), NuPAGE™ Sample Reducing Agent (10X; Cat. No. NP0009), NuPAGE™ MES SDS Running Buffer (20X; Cat. No. NP0002), NuPAGE™ 4 to 12%, Bis-Tris, 1.0 mm, Mini Protein Gel, 10-well (Cat. No. NP0321BOX), NuPAGE™ Transfer Buffer (20X; Cat. No. NP0006), Western Blotting Filter Paper, 0.83 mm thick, 8 × 13.5 cm (Cat. No. 84784), Pierce™ 20X TBS Tween™ 20 Buffer (Cat. No. 28360), Blocker™ BSA (10X) in PBS (Cat. No. 37525), Rabbit polyclonal anti-iNOS antibody (Cat. No. PA3-030A), Rabbit polyclonal anti-GAPDH antibody (Cat. No. PA1-987), Goat anti-Rabbit IgG (H + L) secondary antibody, horseradish peroxidase (HRP)-conjugated (Cat. No. 31460), and Pierce™ ECL Western Blotting Substrate (Cat. No. 32106) were all obtained from Thermo Fisher Scientific (Waltham, MA, USA). HO-1 and OSGIN1 mRNA primers were purchased from Synbio Technologies (Monmouth Junction, NJ, USA). Cell Lysis Buffer (10X; Cat. No. 9803S), Protease Inhibitor Cocktail (100X; Cat. No. 5871), and Prestained Protein Marker, Broad Range (11–190 kDa; Cat. No. 13953S) were purchased from Cell Signaling (Danvers, MA, USA). LPS from Escherichia coli 0111: B4 (Cat. No. L2630) was purchased from Sigma-Aldrich (St. Louis, MO, USA). Recombinant Murine IFN-γ (Cat. No. 315-05) was purchased from PeproTech (Cranbuhry, NJ, USA). Phosphate Buffered Saline (1X PBS; Cat. No. BE17-516F) was purchased from Lonza-Bioscience (Basel, Switzerland). Sulforaphane (Cat. No. 10496) was purchased from Cayman Europe Oü (Tallinn, Estonia). QiAzol lysis reagent (Cat. No. 79306), Nuclease-Free Water (Cat. No. 129114), miScript II RT kit (Cat. No. 218161), miScript SYBR^®^ Green PCR Kit (Cat No. 218073), and miScript primer assays including Hs_RNU6-2_11 (Cat No. MS00033740), Mm_miR-21_2 (Cat. No. MS00011487), Mm-miR-146a*_1 (Cat. No. MS00024220), and Mm_miR-155_1 (Cat. No. MS00001701) were obtained from Qiagen (Hilden, Germany). Dimethyl Sulfoxide (DMSO, research-grade; Cat. No. 20385.02), and Chloroform: Isoamyl alcohol 24:1 (molecular biology grade; Cat. No. 39554.02) were purchased from SERVA (Heidelberg, Germany). Rabbit polyclonal anti-COX-2 antibody (Cat. No. E-AB-70031), Mouse TNF-α (Cat. No. E-EL-M0049), and Mouse IL-6 (Cat. No. E-EL-M0044) Enzyme-Linked Immunosorbent Assay (ELISA) Kits were purchased from Elabscience (Wuhan, China). Acetonitrile and formic acid (LC–MS grade) were obtained from J. T. Baker (Netherlands). MilliQ water was utilized for UPLC-Orbitrap HRMS analysis. All other solvents employed in metabolites profiling were of HPLC–MS grade and were purchased from Sigma Aldrich (St. Louis, MO, USA).

### Fungal material

*T. boudieri* and *T. claveryi*, two desert truffles, were collected from Marsa Matruh, Egypt. Truffles were lyophilized and stored at − 20 °C for further analysis. To specify the concentration of the extracts used in this study, a stock solution was made by dissolving 100 mg of the ethanolic extract in 1 mL of DMSO, resulting in a 100 mg/mL concentration. The solution was then filtered using a 0.2 μm syringe filter to remove impurities. The 1 mg/mL solution was prepared by diluting the stock solution with DMEM medium. A working 100 μg/mL solution was produced by further diluting the 1 mg/mL solution with DMEM medium. The working solution was used to prepare all tested concentrations, ensuring the final DMSO concentration was kept at 0.05% v/v or lower.

### Extraction procedure and sample preparation for UPLC-Orbitrap HRMS analysis

Separately, the freeze-dried truffles were ground in a mortar with liquid nitrogen. The powder (150 mg) was then homogenized for five 20-s periods with 6 mL methanol in a Turrax mixer (11,000 rpm). To avoid heating, each mixing period was separated by 1 min. To remove plant debris, the extracts were vortexed vigorously and centrifuged at 3000× *g* for 30 min. 3 μL of supernatant were used for UPLC-Orbitrap HRMS analysis. Farag's exact parameters of chromatographic conditions and mass spectrometer parameters were followed^32^.

### Identification of metabolites

Compounds were assigned by comparison of their retention times and MS spectral data (accurate mass, isotopic distribution, and fragmentation pattern) in both negative and positive ionization modes with those reported for *Terfezia* species alongside searching the phytochemical dictionary database.

### Cell culture

RAW 264.7 cells (ATCC, TIB-71™) were maintained in DMEM, high glucose supplemented with 10% heat-inactivated FBS, and 1% Pen/Strep (100 units/mL penicillin and 100 µg/mL streptomycin) at 37 °C in a humidified 5% CO2 incubator.

### Cell viability assay

RAW 264.7 cells were seeded at a density of 1 × 10^5^ cells/mL in a 96-well plate and cultured in DMEM, high glucose supplemented with 10% heat-inactivated FBS and 1% Pen/Strep for 4 h at 37 °C in a humidified 5% CO2 incubator. After removing the medium, the cells were treated with increasing concentrations of *T. boudieri* and *T. claveryi* extracts (5, 10, 20, 40, 80, and 160 μg/mL) for 24 h to determine the non-cytotoxic concentrations to be used in the study. The cell viability was measured with the MTT colorimetric assay. After the incubation period, the medium was decanted, and the cells were incubated with serum-free DMEM containing 1 mg/mL of MTT for 2 h at 37 °C in a humidified 5% CO2 incubator. MTT was reduced by NAD(P)H-dependent cellular oxidoreductase enzymes of the metabolically active cells into formazan crystals, which were then dissolved in DMSO. The absorbance of each group was measured at 570 nm using SPECTROstar^®^ Nano microplate reader (BMG LABTECH, Ortenberg, Germany), and the results were presented as the percentage of the control.

In this study, RAW 264.7 cells were stimulated with LPS (100 ng/mL) plus IFN-γ (10 U/mL). Therefore, the cytotoxicity of *T. boudieri* and *T. claveryi* extracts in the presence of LPS/IFN-γ was also evaluated. RAW 264.7 cells were stimulated with LPS/IFN-γ (100 ng/10 U/mL), and co-incubated with the non-cytotoxic concentrations of *T. boudieri* and *T. claveryi* extracts 5, 10, and 20 µg/mL for 24 h in a 96-well plate. Additionally, the cells were treated with vehicle control, which was DMEM medium containing 0.05% v/v DMSO matching the maximum final concentration of DMSO in our treatments. Sulforaphane (SFN) (1 µM) was used as a positive control in all further experiments, so its cytotoxicity on RAW 264.7 cells with or without LPS/IFN-γ was also examined. The cell viability was then measured with MTT, as previously mentioned.

### Nitrite assay

RAW 264.7 cells were seeded at a density of 1 × 10^6^ cells/mL in a 96-well plate and incubated for 4 h. The cells were then stimulated with LPS/IFN-γ (100 ng/10 U/mL) and co-incubated with *T. boudieri* and *T. claveryi* extracts at concentrations of 5, 10, and 20 µg/mL for 24 h at 37 °C in a humidified 5% CO2 incubator. SFN (1 µM) was used as a positive control. The nitrite accumulated in the culture medium was measured as an indicator of NO production using the Griess Reagent Kit by mixing 150 µL of the culture supernatant from each well with 130 µL of deionized water and 20 µL of Griess reagent and incubating at room temperature for 30 min in the dark according to the manufacturer’s instructions. The absorbance of the mixture was measured at 548 nm using a SPECTROstar^®^ Nano microplate reader. The nitrite concentration in each sample was calculated based on a standard curve prepared with NaNO2.

### Quantitative real-time polymerase chain reaction (qPCR)

RAW 264.7 cells were seeded at a density of 1 × 10^6^ cells/mL in a 6-well plate and incubated overnight. The cells were then stimulated with LPS/IFN-γ (100 ng/10 U/mL) and co-incubated with *T. boudieri* and *T. claveryi* extracts at concentrations of 5, 10, and 20 µg/mL for 6 h at 37 °C in a humidified 5% CO2 incubator. SFN (1 µM) was used as a positive control. Total RNA was extracted from the cells using QiAzol lysis reagent and used for cDNA synthesis. The cDNAs were synthesized from the total RNA using Thermo Scientific™ RevertAid™ First Strand cDNA Synthesis Kit for mRNA and miScript^®^ II RT Kit for the microRNA (miRNA). The qPCR analyses were then performed using PowerUp™ SYBR™ Green Master Mix to quantify the mRNA expression of iNOS, COX-2, TNF-α, IL-6, HO-1, OSGIN1, and GAPDH (as a housekeeping gene). Furthermore, the miScript SYBR^®^ Green PCR Kit was used to quantify the miRNA expression of miR-21, miR-146a, and miR-155, and RNU6-2 (as a housekeeping gene). The qPCR analyses were conducted using ABI Prism 7500 system (Applied Biosystems). The relative gene expression levels were determined by the 2^−ΔΔCT^ method. The final results were expressed as the fold change of target gene expression in a target sample (treated sample) relative to a reference sample (untreated control), normalized to a reference gene: GAPDH (for mRNA) or RNU6-2 (for miRNA) as the following: fold change = 2^−ΔΔCT^, while ΔΔCT = ΔCT (treated sample) − ΔCT (untreated control), and ΔCT = CT (target gene) − CT (reference gene). The mRNA primers used in this study were designed using NCBI Primer-BLAST and are listed in Table 1. The miScript primer assays (miRNA-specific forward primers) were purchased from Qiagen. The miScript universal primer (miRNA reverse primer) was provided in the miScript SYBR^®^ Green PCR Kit. It was applied to all reactions allowing the detection of miRNAs in combination with miScript primer assays. Primer assays used in this study are listed in Table 2.

**Table 1:** Primer sequences used for the qPCR analysis.

Target gene	Forward primer (5′–3′)	Reverse primer (5′–3′)
iNOS	GGAACCTACCAGCTCACTCTGG	TGCTGAAACATTTCCTGTGCTGT
COX-2	CTCACGAAGGAACTCAGCAC	GGATTGGAACAGCAAGGATTTG
TNF-α	GAACTCCAGGCGGTGCCTAT	TGAGAGGGAGGCCATTTGGG
IL-6	GATGCTACCAAACTGGATATAATCAG	CTCTGAAGGACTCTGGCTTTG
HO-1	CACAGATGGCGTCACTTCGTC	GTGAGGACCCACTGGAGGAG
OSGIN1	CGGTGACATCGCCCACTAC	GCTCGGACTTAGCCCACTC
GAPDH	CTTTGTCAAGCTCATTTCCTGG	TCTTGCTCAGTGTCCTTGC

**Table 2:** Primer assays used for the qPCR analysis.

miRNA	Product Information
miR-21	Mm_miR-21_2 miScript Primer Assay
miR-146a	Mm-miR-146a*_1 miScript Primer Assay
miR-155	Mm_miR-155_1 miScript Primer Assay
RNU6-2	Hs_RNU6-2_11 miScript Primer Assay

### Enzyme-linked immunosorbent assay (ELISA) for TNF-α and IL-6

RAW 264.7 cells were seeded at a density of 1 × 10^6^ cells/mL in a 6-well plate and incubated overnight. The cells were then stimulated with LPS/IFN-γ (100 ng/10 U/mL) and co-incubated with *T. boudieri* and *T. claveryi* extracts at concentrations of 5 and 20 µg/mL for 24 h at 37 °C in a humidified 5% CO2 incubator. SFN (1 µM) was used as a positive control. After incubation, the supernatants were collected, centrifuged at 1000× *g* for 20 min at 4 °C, and then stored at − 80 °C. The samples were subsequently analyzed for TNF-α and IL-6 proteins. The absorbance was measured at 450 nm using SPECTROstar^®^ Nano microplate reader. The concentration of the mouse TNF-α and IL-6 proteins in each sample was calculated by comparing the absorbance of the samples to the standard curve.

### Western blotting

RAW 264.7 cells were seeded at a density of 1 × 10^6^ cells/mL in a 6-well plate and incubated overnight. The cells were then stimulated with LPS/IFN-γ (100 ng/10 U/mL) and co-incubated with *T. boudieri* and *T. claveryi* extracts at concentrations of 20 µg/mL for 24 h at 37 °C in a humidified 5% CO2 incubator. SFN (1 µM) was used as a positive control. Total protein was extracted from the cells using an ice-cold cell lysis buffer containing a protease inhibitor cocktail. The protein concentration for each cell lysate was measured using Pierce™ BCA Protein Assay Kit. For western blotting, an equal amount of protein (10 μg) was loaded into each lane of the NuPAGE™ 4 to 12%, Bis–Tris Gel, and the Prestained Protein Marker. The gel ran into 1× NuPAGE™ MES SDS Running Buffer for 35 min at 200 Volts. The gel electrophoresis was conducted using the XCell SureLock™ Mini-Cell (Thermo Fisher Scientific, MA, USA). After electrophoresis, the protein was transferred into a PVDF membrane through an electrotransfer process which was performed for 60 min at 30 Volts to transfer the protein from the gel to the PVDF membrane via 1× NuPAGE™ Transfer Buffer using the XCell II™ Blot Module (Thermo Fisher Scientific, MA, USA). After blocking in 5% Blocker™ BSA for 1 h at room temperature, the membrane was incubated overnight at 4 °C with primary antibodies; Rabbit polyclonal anti-iNOS antibody (1: 2000), Rabbit polyclonal anti-COX-2 antibody (1: 1000), and Rabbit polyclonal anti-GAPDH antibody (1: 2500). The membrane was then incubated for 1 h at room temperature with Goat anti-Rabbit IgG (H + L) secondary antibody, horseradish peroxidase (HRP)-conjugated (1: 15,000). Proteins were visualized with the Pierce™ ECL Western Blotting Substrate using ChemiDoc MP Imaging System (Bio-Rad Laboratories, CA, USA). The density of western blotting bands was quantified using the ImageLab software (Bio-Rad Laboratories). The density of the protein bands was normalized to that of GAPDH. The density values were relatively expressed to the average value for the untreated control group, which was designated as 1.0.

### Statistical analysis

Data are expressed as mean ± standard error of the mean (SEM) for the indicated number of independently performed experiments. Statistical analysis was performed using SigmaPlot Version 14.0 (Systat Software, Inc., San Jose, CA, USA). Statistical significance between multiple groups was calculated by one-way analysis of variance (ANOVA) followed by Student–Newman–Keuls post-hoc test, where *P* value < 0.05 was considered statistically significant. All values obtained from the Griess assay, ELISA, and BCA assay were subjected to linear regression analysis.

## Results

### Metabolites profiling of *T. boudieri* and *T. claveryi*

To assess for differences in the two desert truffles' metabolite composition, a non-targeted metabolite profiling of their ethanolic extracts was employed. The secondary metabolites were tentatively identified in the two desert truffles, *T. boudieri* and *T. claveryi, *via* high-resolution* UPLC-Orbitrap HRMS analysis. A total of 87 different metabolites belonging to various metabolite classes were annotated, including 21 amino acids and sugars, 11 alkaloids, 2 amides, 23 fatty acids/esters, 5 sterols, 4 phenolic, and 21 miscellaneous peaks, as listed in Table 3. Provided below are details for the identification of each metabolites class.

**Table 3:** Metabolites composition of *T. boudieri* and *T. claveryi* analyzed via UPLC-MS.

No	Proposed structure	Molecular formula	Error in ppm	MS^2^	*T. boudieri*	*T. claveryi*	M − H	M + H	RT
*Amino acids and carbohydrates*									
1	N, N′-Di-α-hexosyl-butanediamine	C_16_H_32_N_2_O_10_	4.4	342, 234	+			413.211	0.743
2	Choline Glutamate	C_10_H_22_N_2_O_5_	6.2	233, 216, 198	+			251.159	0.777
3	Eletefine	C_19_H_19_NO_5_	− 8.5	324, 306, 288	+			342.134	0.811
4	N-Methyl-L-glutamic acid	C_6_H_11_NO_4_	5.4	146,127	+			162.076	0.849
5	N-Glucosyl arginine	C_12_H_24_N_4_O_7_	7.3	319, 175	+			337.172	0.856
6	Arginine	C_6_H_14_N_4_O_2_	5.5	158	+			175.118	0.859
7	Xylonic acid	C_5_H_10_O_6_	1.9	147, 129, 87, 75	+		165.041		0.946
8	Unknown amino sugar	C_9_H_17_NO_7_	5.5	234, 216, 170	+	+		252.108	0.955
9	Sucrose	C_12_H_22_O_11_	− 0.1	162		+	341.109		0.994
10	Fructosyl valine	C_11_H_21_NO_7_	0.1	118, 262, 126	+			280.137	1.014
11	N-Fructosyl pyroglutamate	C_11_H_17_NO_8_	− 1.3	128, 170, 200, 243, 272	+	+	290.088		1.021
12	N-fructosyl isoleucine	C_12_H_23_NO_7_	0.1	276, 258, 230, 212, 86	+	+		294.155	1.036
13	Pyroglutamic acid	C_5_H_7_NO_3_	0.3			+	1,280,353	130.05	1.463
14	Unknown amino sugar	C_16_H_23_NO_10_	− 1.3	244, 287, 306	+	+	388.125		1.864
15	N-Fructosyl phenylalanine	C_15_H_21_NO_7_	4.7	310, 292, 264, 178, 166	+	+		328.137	2.682
16	N-acyl homoserine lactone	C_9_H_16_NO_4_	3.4	186, 168, 99	+			202.107	2.74
17	Unknown amino sugar	C_14_H_21_NO_9_	− 1.9	189, 273		+	346.111		3.168
18	Pipecolate	C_6_H_10_NO_2_	3	113	+			128.071	3.275
19	Stachydrine	C_7_H_14_NO_2_	3.8		+			144.102	3.911
20	N-Acetyl-L-leucine	C_8_H_15_NO_3_	-4.7	130		+	172.099		5.232
21	N-Acetylaspartic acid	C_14_H_25_NO_5_	− 0.2	214		+	286.166		11.748
*Alkaloids*									
22	Vincapyridine C	C_12_H_16_NO_4_	4.4	220, 179	+	+		238.107	5.273
23	4-Hydroxy-2,2,6,6-tetramethylpiperidine-1-oxyl	C_9_H_18_NO_2_	− 0.1	85		+		172.133	6.202
24	Puniceusine F	C_14_H_16_NO_4_	2.6	244, 216,192		+		262.107	6.38
25	Hyrtiosine A	C_10_H_9_NO_3_	4.3	176, 164	+			192.066	6.418
26	Piperlongumine	C_17_H_19_NO_5_	1.7	212.1056		+	316.119	318.134	6.578
27	augustine N-oxide	C_17_H_20_NO_5_	3.1	288, 258, 242		+		318.134	8.918
28	Fabiocinine	C_15_H_14_NO_3_	4.1	210, 178, 105	+			256.097	11.654
29	Tersone G	C_14_H_12_NO_2_	2.8	208, 172, 148, 105	+			226.086	12.186
30	Norargemonine	C_20_H_22_NO_4_	3.6	294, 266	+			340.153	13.641
31	2-methyl-6-(9-phenylnonyl) piperidine-3,4-diol	C_21_H_36_NO_2_	3.3	236, 220	+			334.274	14.17
32	17,20-dihydroxybuxadine-M	C_25_H_42_NO_2_	1.8	238, 222	+			388.321	15.838
*Amides*									
33	Dodecanamide	C_12_H_26_NO	− 1.4		+			200.201	14.79
34	Octadecenoic acid amide	C_18_H_35_NO	2	247, 212, 163, 97		+		282.279	17.6
*Fatty acids/esters*									
35	Hydroxytetradecanedioic acid	C_14_H_26_O_5_	− 2			+	273.171		10.415
36	Octadecenedioic acid	C_18_H_32_O_4_	− 0.7	311, 293	+	+	311.222	313.237	11.467
37	Hydroxyoctadecanedioic acid	C_18_H_34_O_5_	− 0.1	229, 211	+	+	329.233		11.666
38	Dodecanedioic acid	C_12_H_22_O_4_	0.3	229, 211, 167	+		229.145		11.979
39	Octadecenedioic acid	C_18_H_33_O_4_	3.8	295, 277		+		313.236	12.129
40	Trihydroxy trioxo docosanoic acid	C_22_H_37_O_8_	4.2	411, 393, 293	+			429.248	12.606
41	Unknown fatty acyl amide	C_23_H_44_NO_3_	3	357, 340, 277		+		382.332	12.723
42	Hexadecanedioic acid	C_16_H_30_O_4_	− 0.7		+		285.207		13.137
43	Octadecanedioic acid	C_18_H_34_O_4_	0.3	295, 277, 183, 129	+	+	313.238		13.528
44	Hydroxy-octadecanoic acid	C_18_H_35_O_4_	3.3	279, 261, 243	+	+		315.253	13.569
45	Hydroxy-octadecenoic acid	C_18_H_33_O_3_	3.1	261, 243	+			279.231	14.064
46	Octadecatetraenoic acid	C_18_H_28_O_2_	3.3	231, 176, 163		+		277.215	14.181
47	Unknown oxylipid	C_22_H_40_O_7_	− 0.2		+		415.27		14.406
48	Hydroxy-octadecadienoic acid	C_18_H_32_O_3_	− 0.9	295, 277, 195	+	+	295.228		15.023
49	Hydroxy-octadecatrienoic acid	C_18_H_30_O_3_	0.4		+		293.212	295.227	15.288
50	Hydroxyoctadecanoic acid	C_18_H_36_O_3_	1.3		+		299.258		16.119
51	Oxo-octadecadienoic acid	C_18_H_28_O_3_	0.8	275, 223, 205, 187	+			293.21	16.522
52	Octadecatrienoic acid	C_18_H_30_O_2_	− 0.5	261, 223, 209, 173	+		277.218	279.232	16.812
53	Hydroxyhexadecanoic acid	C_16_H_32_O_3_	1		+	+	271.228		16.992
54	linoleoyl glycerol	C_21_H_38_O_4_	1.8	337, 263, 245	+	+		355.284	16.962
55	Hexadecenoic acid	C_16_H_30_O_2_	0.4		+		253.217		17.239
56	Linoleic acid	C_18_H_32_O_2_	− 0.1	263, 245	+	+	279.233	281.248	17.46
57	Hexadecanoic Acid	C_16_H_32_O_2_	− 0.4	229, 173	+		255.233	257.248	17.646
*Sterols*									
58	Hydroxystigmast-4-en-	C_29_H_48_O_2_	3.1	297, 233		+		429.372	15.852
	3-one								
59	dankasterone A	C_28_H_40_O_3_	3.3	407, 297, 165		+		425.304	15.976
60	Matsutoic acid	C_18_H_30_O_3_	4.4	277, 259, 198, 180		+		295.224	16.202
61	Unknown sterol	C_19_H_30_O_3_	2.4	289, 251		+		307.227	16.367
62	Paxisterol	C_28_H_42_O_4_	2.3	341		+		443.316	17.034
*Phenolic compounds*									
63	Hydroquinone-glucose-O-hexoside	C_12_H_15_O_7_	4.1	161, 109, 101	+		271.081		1.624
64	Scopoletin	C_10_H_8_O_4_	3.7		+		191.034		10.757
65	Flavonol 3-O-petosyl hexoside	C_26_H_28_O_12_	− 5.7	487, 443	+		531.154		11.667
66	isomyrtucommulone B	C_24_H_30_O_6_	2.8	281, 119		+		415.212	13.786
*Miscellaneous*									
67	Malic acid	C_4_H_6_O_5_	− 1.5	115, 87,71, 87	+	+	133.014		1.011
68	Unknown hexose	C_6_H_10_O_6_	− 0.6	129, 99, 89, 85, 75	+	+	177.041		1.028
69	Citric acid	C_6_H_8_O_7_	− 0.6	129, 111, 87		+	191.02		1.028
70	Succinic acid	C_4_H_6_O_4_	− 0.8	117, 99, 73	+		117.019		1.555
71	Citramalic acid	C_5_H_8_O_5_	3.2	147, 103, 87		+	147.029		1.597
72	isoquinoline-1,4-diol	C_10_H_13_NO_2_	4.1	103, 89	+			162.055	3.327
73	Menisdaurin D	C_14_H_20_N13O_7_	2.3	225, 180	+			314.123	3.543
74	Homotyrosine methyl ether	C_11_H_15_NO_3_	4.2	192, 96	+			210.113	3.774
75	Dihydroxybenzenebutanoic acid O hexoside	C_16_H_22_O_9_	5.3	313	+		357.117		4.677
76	Cinnamic acid	C_9_H_8_O_2_	2.9			+	147.045		5.761
77	Unknown nitrogenous	C_12_H_17_NO_3_	0.2	128, 89	+	+		224.128	6.99
78	Unknown aromatic	C_14_H_10_O_3_	6.2	124	+		225.054		7.553
79	Unknown amino sugar	C_12_H_19_NO_6_	4	144		+	272.113		7.567
80	Phenylacetic acid	C_8_H_8_O_2_	1.3		+		135.041		7.639
81	unknown terpenoid	C_16_H_18_O_5_	4.4	181, 163		+		291.123	7.765
82	1-(1-b-Glucopyranosyl)-1H-indole-3-carbaldehyde	C_15_H_17_NO_6_	− 8.7	289, 261		+	306.101		12.179
83	Amino-octadecene-triol	C_18_H_38_NO_3_	4	298, 280	+			316.283	12.723
84	Unknown terpenoid	C_19_H_25_O_5_	4.2	289, 247, 203	+			333.17	13.042
85	Nitrogenous lipid	C_18_H_29_NO_3_	3	290, 210, 192		+		308.221	14.666
86	LysoDGTSA 16:0	C_26_H_51_NO_6_	2.1	456, 236		+		474.377	15.593
87	unknown diterpene	C_20_H_34_O_2_	1.8	277, 261, 243, 219, 205, 187, 173, 163, 109, 95		+		307.263	16.9

### Amino acids and sugars

Truffles are typical edible fungi that are rich in nutrients, such as carbohydrates and amino acids, as revealed using LCMS analysis. Analysis showed the presence of several sugars and amino sugars like peak 10 with [M + H]^+^at *m/z* 280.1372 (C_11_H_21_NO_7_), peak 11 with [M − H]^−^ at *m/z* 290.0881 (C_11_H_17_NO_8_), peak 12 with [M + H]^+^ at *m/z* 294.1547 (C_12_H_23_NO_7_) were annotated as fructosyl valine, *N*-fructosyl pyroglutamate and *N*-fructosyl isoleucine^33^, respectively.

Twenty-one amino acids and sugars were detected in the extracts of the two desert truffles, with *T. boudieri* containing more identified amino acids (16 peaks were annotated) and *T. claveryi* containing only 10 peaks. (Peaks 8, 11, 12, 14, 15 and 16) were detected in both species. Identification of amino acids was mostly based on detection of nitrogen in their predicted formula and confirmed using tandem MS. For example, peak 5 showed [M + H]^+^ at *m/z* 337.1718 (C_12_H_24_N_4_O_7_) and yielded fragment ions at 319, 175 was annotated as *N*-Glucosyl arginine. Peak 6 showed [M + H]^+^ at *m/z* 175.1180 (C_6_H_14_N_4_O_2_) and yielded fragment ions at 158 was annotated as arginine. Peak 11 showed [M − H]^−^ at *m/z* 290.0881 (C_11_H_17_NO_8_) and yielded fragment ions at *m/z* 128, 200, 272 was annotated as *N*-Fructosyl pyroglutamate. Peak 12 with an [M + H]^+^ at *m/z* 294.1547 (C_12_H_23_NO_7_) and fragment ions at *m/z* 276, 258, 230, 212, 86.0968; was annotated as *N*-fructosyl isoleucine^33^, while peak 15 with an [M + H]^+^ at *m/z* 328.1369 (C_15_H_21_NO_7_)^+^ and fragment ions at *m/z* 310, 292, 264, 178, 166; was annotated as *N*-Fructosyl phenylalanine^34^.

### Alkaloids and nitrogenous compounds

MS spectral analysis also revealed the presence of nitrogenous compounds or alkaloids typically derived from amino acids. Based on their even high-resolution masses and improved response in positive ionization mode, 11 alkaloids (peaks 22–32) were annotated. Peak 24, for example, was annotated as puniceusine F^35^, with the molecular formula (C_14_H_16_NO_4_)^+^_._ Furthermore, peak 32, (C_25_H_42_NO_2_)^+^ was annotated as dihydroxybuxadine-M. *T. boudieri* contains 7 alkaloids, whereas *T. claveryi* contains only 5 alkaloids, with Vincapyridine C being the only alkaloid present in both.

Truffles appeared to be enriched in nitrogenous compounds in addition to alkaloids, several nitrogenous compounds sub-classified as amides were identified. Peaks 33 and 34 showed [M + H]^+^ *m/z* at 200.2009 C_12_H_26_NO^+^ and 282.2785 C_18_H_35_NO^+^, annotated as dodecanamide and octadecenoic acid amide, respectively. The defining features of the monounsaturated fatty acid amide fragmentation patterns were based on losses of [M + H-17] and [M + H-35] product ion peaks observed in octadecenoic acid amide fragmentation, indicating the loss of ammonia and water from the carboxamide head group^36^.

### Fatty acids/esters

In the late elution region of the chromatogram, several peaks were observed. MS/MS spectra (Rt 10–18 m min) in the extracts of *T. boudieri* and *T. claveryi* revealed the presence of several fatty acids annotated by high-resolution masses and much higher response in negative ionization mode as major peaks. Octadecatrienoic acid (linolenic acid, peak no. 52) and octadecadienoic acid (linoleic acid, peak no. 56) were readily identified based on their high-resolution masses *m/z* 277.2175 and 279.2330 with predicted molecular formulas C_18_H_30_O_2_^-^, and C_18_H_32_O_2_^-^, respectively. A mass difference of 2 amu between the two peaks indicates the presence of an additional double bond.

MS/MS spectra also revealed several mono, di, and trihydroxy fatty acid conjugates assigned from their high-resolution masses and predicted formulas (35, 37, 40, 44, 45, 48, 49, 50, and 53) (Table 3). Negative ion MS was also revealed for several hydroxylated fatty acids. The major hydroxy fatty acids identified in both *Terfezia species* were annotated as hydroxy octadecanedioic acid (37), hydroxy-octadecadienoic acid (48), and hydroxy-octadecatrienoic acid (49) (329.2330, 295.2280, and 293.2121, respectively).

### Sterols

In *T. claveryi*, five sterols with higher response in positive ionization mode were identified, including peak 58, showed [M + H]^+^ at *m/z* 429.3721 (C_29_H_48_O_2_)^+^ and yielded fragment ions at *m/z* 297, 233 was annotated as hydroxystigmast-4-en-3-one^37^. Peak 59 showed [M + H]^+^ at *m/z* 425.3044 (C_28_H_40_O_3_)^+^ and yielded fragment ions at *m/z* 407, 297, and 165, which was annotated as dankasterone A^38^. No sterols were detected in *T. boudieri*.

### Phenolic compounds

The MS/MS spectra revealed a few phenolic compounds. Three peaks were detected in *T. boudieri,* peak 63 with [M-H]^-^ at *m/z* 271.0812 (C_12_H_15_O_7_)^−^, peak 64 with [M − H]^−^ at m/z 191.0343 (C_10_H_8_O_4_)^−^, peak 65 with [M − H]^−^ at m/z 531.1538 (C_26_H_28_O_12_)^−^ were annotated as hydroquinone-glucose-*O*-hexoside, scopoletin, and flavonol 3-*O*-petosyl hexoside, respectively. Only one peak 66 was identified in *T. claveryi* showed [M + H]^+^ at *m/z* 415.2115 (C_24_H_30_O_6_)^+^ was annotated as isomyrtucommulone B.

### Miscellaneous

6 Annotated peaks belonged to organic acids identified in peaks 67, 69, 70, 71, 76, and 80. peak 67 showed [M − H]^−^ at m/z 133.0144 (C_4_H_6_O_5_)^−^ and yielded fragment ions at 115, 87,71, 87 was annotated as Malic acid. peak 69 showed [M − H]^−^ at m/z 191.0198 (C_6_H_8_O_7_) and yielded fragment ions at 129, 111, and 87 annotated as Citric acid. Peak 70 showed [M − H]^−^ at m/z 117.0190 (C_4_H_6_O_4_)^−^ and yielded fragment ions at 117, 99, and 73 were annotated as Succinic acid. Few terpenes were detected in two types of truffles under study represented in peak 81 with [M + H]^+^ at m/z 291.1227 (C_16_H_18_O_5_)^+^, peak 84 with [M + H]^+^ at m/z 333.1697 (C_19_H_25_O_5_)^+^, peak 87 with [M + H]^+^ at m/z 307.2627 (C_20_H_34_O_2_)^+^ were annotated as unknown terpenoids.

### Effect of *T. boudieri* and *T. claveryi* extracts on the viability of RAW 264.7 cells

**Figure 1:**
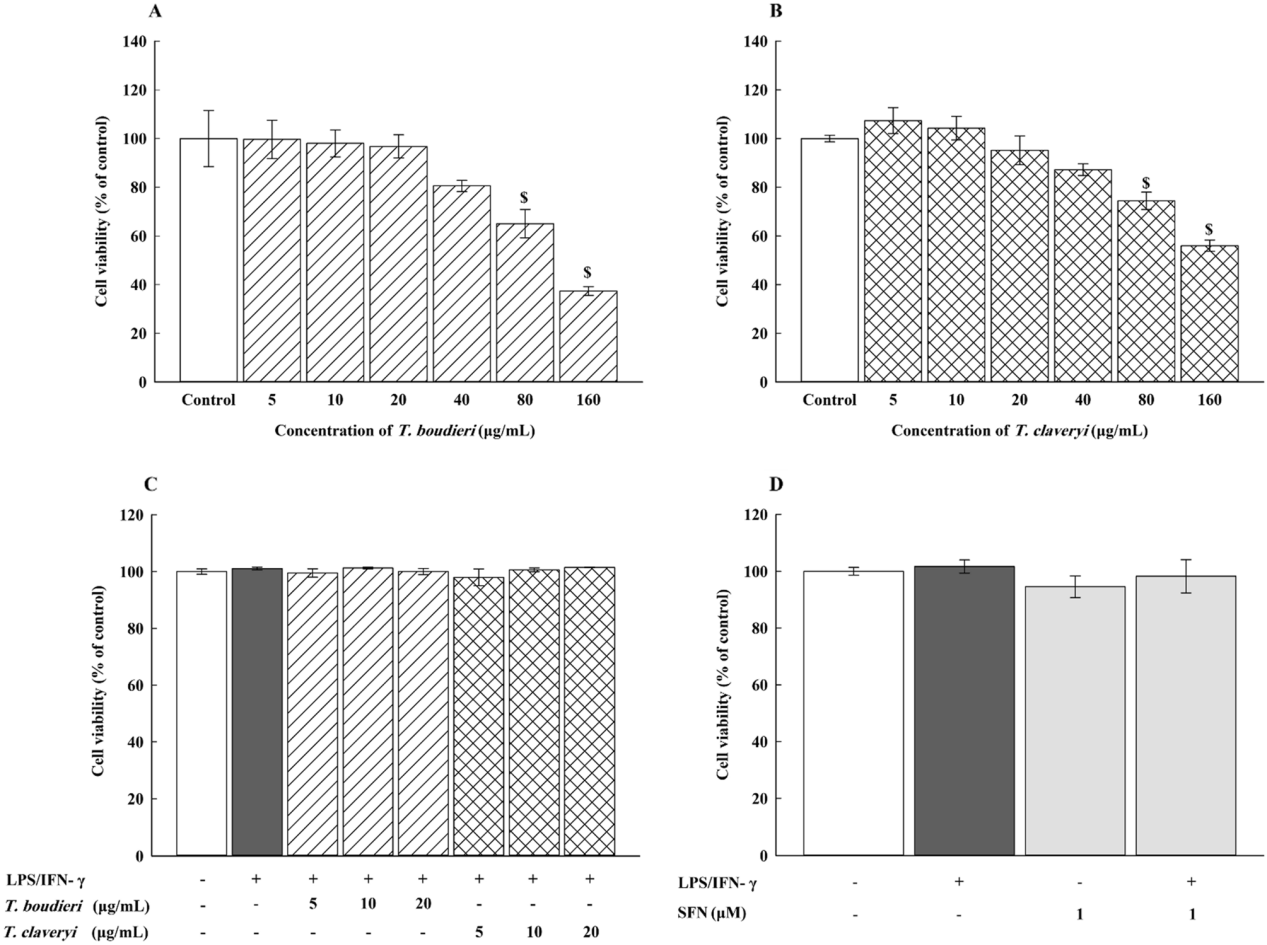
Effect of *T. boudieri* and *T. claveryi* extracts on the viability of RAW 264.7 cells. RAW 264.7 cells were separately incubated with increasing concentrations (5–160 μg/mL) of *T. boudieri* (**A**) and *T. claveryi* (**B**) extracts or with LPS/IFN-γ (100 ng/10 U/mL) (**C**), and SFN (1 µM) as a positive control (**D**) for 24 h. Cell viability was measured using an MTT assay. Data are expressed as a percentage of control (100%) ± SEM (n = 8). Statistical significance was calculated by one-way ANOVA followed by Student–Newman–Keuls post-hoc test. $*P* < 0.05 versus control.

Before examining the effect of *T. boudieri* and *T. claveryi* extracts on LPS/IFN-γ-induced inflammation in RAW 264.7 cells, an MTT assay was performed to determine the optimal concentrations for use in the study. RAW 264.7 cells were incubated with increasing concentrations of *T. boudieri* and *T. claveryi* extracts (5–160 μg/mL) in the presence or absence of LPS/IFN-γ (100 ng/10 U/mL) for 24 h. SFN (1 µM) was used as a positive control. Both extracts did not significantly affect the cell viability at concentrations below 40 μg/mL compared to the untreated control. However, cell viability was decreased by approximately 63% with *T. boudieri* and 46% with *T. claveryi* when the concentration was increased to 160 μg/mL, revealing that *T. boudieri* has more cytotoxic effect on RAW 264.7 cells (Fig. 1A,B). When the cells were co-incubated with LPS/IFN-γ, similar results were observed with no cytotoxicity at concentrations of 5, 10, and 20 µg/mL for both extracts (Fig. 1C). SFN (1 µM) alone and combined with LPS/IFN-γ did not show any cytotoxicity on RAW 264.7 cells (Fig. 1D). Therefore, *T. boudieri* and *T. claveryi* extracts were used at concentrations of 5, 10, 20 μg/mL in all subsequent experiments to investigate their effects on inflammation induced by LPS/IFN-γ in RAW 264.7 cells.

### Effect of *T. boudieri* and *T. claveryi* extracts on nitrite production in LPS/IFN-γ-stimulated RAW 264.7 cells

**Figure 2:**
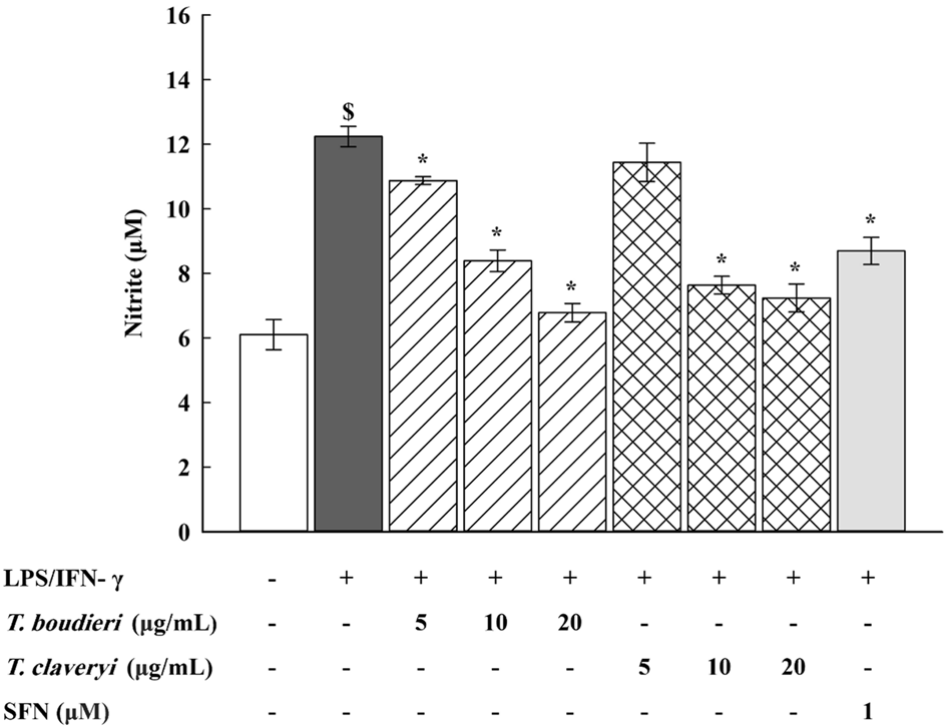
Effect of *T. boudieri* and *T. claveryi* extracts on nitrite production in LPS/IFN-γ-stimulated RAW 264.7 cells. RAW 264.7 cells were stimulated with LPS/IFN-γ (100 ng/10 U/mL) and co-incubated with *T. boudieri* and *T. claveryi* extracts at concentrations of 5, 10, and 20 µg/mL for 24 h. SFN (1 µM) was used as a positive control. Nitrite levels were analyzed by the Griess Reagent Kit. Data are expressed as mean ± SEM (n = 8). Statistical significance was calculated by one-way ANOVA followed by Student–Newman–Keuls post-hoc test. $*P* < 0.05 versus control. *P < 0.05 versus LPS/IFN-γ.

To investigate the anti-inflammatory effects of *T. boudieri* and *T. claveryi* extracts on nitrite production, RAW 264.7 cells were treated with *T. boudieri* and *T. claveryi* extracts at concentrations of 5, 10, and 20 µg/mL in the presence of LPS/IFN-γ (100 ng/10 U/mL) for 24 h. SFN (1 µM) was used as a positive control. The nitrite levels in the cell culture medium were measured using the Griess Reagent Kit. As shown in Fig. 2**,** nitrite levels exhibited a substantial increase of 200% compared to the untreated control after stimulating cells with LPS/IFN-γ. However, treatment with both *Terfezia* extracts inhibited nitrite production in LPS/IFN-γ-stimulated cells in a dose-dependent manner. *T. boudieri* extract at concentrations of 5, 10, and 20 µg/mL significantly inhibited nitrite production by 11, 31, and 45%, respectively, and *T. claveryi* at concentrations of 10 and 20 µg/mL showed statistically significant inhibition of nitrite production by 38 and 41%, respectively. SFN (1 µM) also decreased nitrite levels by 29%.

### Effect of *T. boudieri* and *T. claveryi* extracts on the mRNA expression of iNOS in LPS/IFN-γ-stimulated RAW 264.7 cells

**Figure 3:**
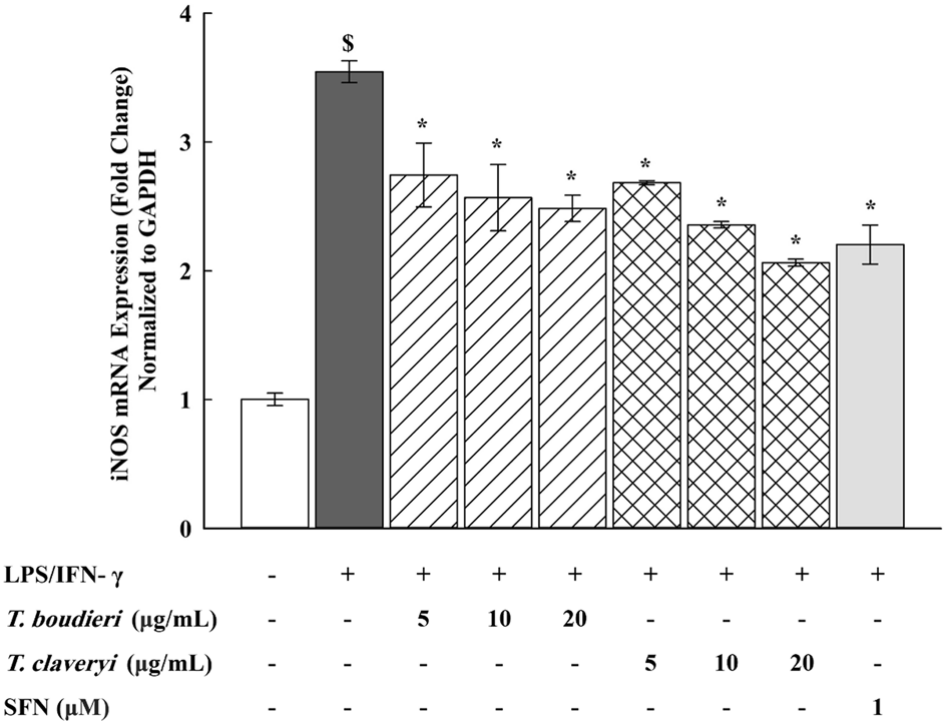
Effect of *T. boudieri* and *T. claveryi* extracts on the mRNA expression of iNOS in LPS/IFN-γ-stimulated RAW 264.7 cells. RAW 264.7 cells were stimulated with LPS/IFN-γ (100 ng/10 U/mL) and co-incubated with *T. boudieri* and *T. claveryi* extracts at concentrations of 5, 10, and 20 µg/mL for 6 h. SFN (1 µM) was used as a positive control. iNOS mRNA expression level was analyzed by qPCR. Data are expressed as mean ± SEM (n = 3). Statistical significance was calculated by one-way ANOVA followed by Student–Newman–Keuls post-hoc test. $*P* < 0.05 versus control. *P < 0.05 versus LPS/IFN-γ.

To investigate whether the reduction of nitrite production following treatment with *Terfezia* extracts was due to suppression of iNOS gene expression, RAW 264.7 cells were treated with *T. boudieri* and *T. claveryi* extracts at concentrations of 5, 10, and 20 µg/mL in the presence of LPS/IFN-γ (100 ng/10 U/mL) for 6 h. SFN (1 µM) was used as a positive control. iNOS mRNA expression was then determined using qPCR. As shown in Fig. 3, LPS/IFN-γ remarkably upregulated the mRNA expression of iNOS by 354% compared to the untreated control. Nevertheless, when the LPS/IFN-γ-stimulated cells were co-treated with *Terfezia* extracts, *T. boudieri* at concentrations of 5, 10, and 20 µg/mL dose-dependently suppressed the upregulation of iNOS mRNA expression by 23, 28, and 30%, respectively, and *T. claveryi* extract at concentrations of 5, 10, and 20 µg/mL downregulated iNOS mRNA expression by 24, 34, and 42%, respectively, in a dose-dependent manner. SFN (1 µM) also inhibited the iNOS mRNA expression by 38%. These results indicate that *Terfezia* extracts decreased nitrite production in LPS/IFN-γ-stimulated macrophages by downregulating iNOS transcription.

### Effect of *T. boudieri* and *T. claveryi* extracts on the mRNA expression of COX-2 in LPS/IFN-γ-stimulated RAW 264.7 cells

**Figure 4:**
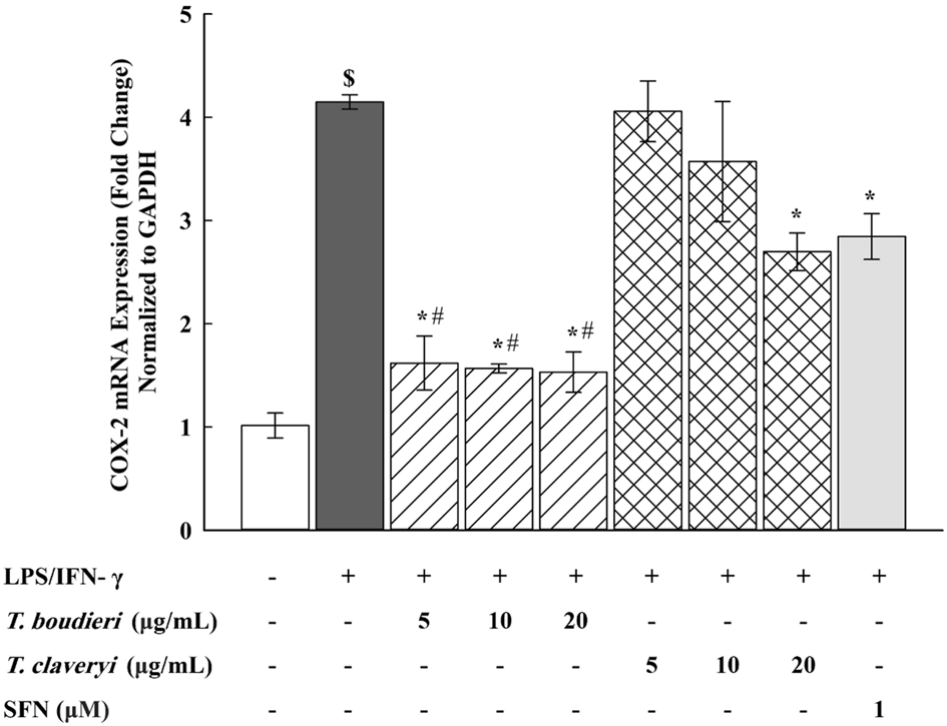
Effect of *T. boudieri* and *T. claveryi* extracts on the mRNA expression of COX-2 in LPS/IFN-γ-stimulated RAW 264.7 cells. RAW 264.7 cells were stimulated with LPS/IFN-γ (100 ng/10 U/mL) and co-incubated with *T. boudieri* and *T. claveryi* extracts at concentrations of 5, 10, and 20 µg/mL for 6 h. SFN (1 µM) was used as a positive control. COX-2 mRNA expression level was analyzed by qPCR. Data are expressed as mean ± SEM (n = 3). Statistical significance was calculated by one-way ANOVA followed by Student–Newman–Keuls post-hoc test. $*P* < 0.05 versus control. *P < 0.05 versus LPS/IFN- γ. #*P* < 0.05, *T. boudieri* at any given concentration versus *T. claveryi* at the corresponding concentration.

To examine the suppressive effect of *Terfezia* extracts on the COX-2 mRNA expression, RAW 264.7 cells were treated with *T. boudieri* and *T. claveryi* extracts at concentrations of 5, 10, and 20 µg/mL in the presence of LPS/IFN-γ (100 ng/10 U/mL) for 6 h. SFN (1 µM) was used as a positive control. COX-2 mRNA expression was then determined using qPCR. Figure 4 shows that the mRNA expression of COX-2 was significantly induced by 409% compared to the untreated control following stimulation with LPS/IFN-γ. However, when the LPS/IFN-γ-stimulated cells were co-treated with *Terfezia* extracts, *T. boudieri* strongly downregulated the mRNA expression of COX-2 by approximately 60% (5–20 µg/mL), and *T. claveryi* extract at only 20 µg/mL significantly downregulated the mRNA expression of COX-2 by 35%. Significant differences were also observed in the inhibitory effects of both *Terfezia* extracts on the COX-2 mRNA expression, with *T. boudieri* being statistically significant compared to *T. claveryi* at 5, 10, and 20 µg/mL. SFN (1 µM) also inhibited the COX-2 mRNA expression by 31%.

### Effect of *T. boudieri* and *T. claveryi* extracts on the mRNA expression of TNF-α and IL-6 in LPS/IFN-γ-stimulated RAW 264.7 cells

**Figure 5:**
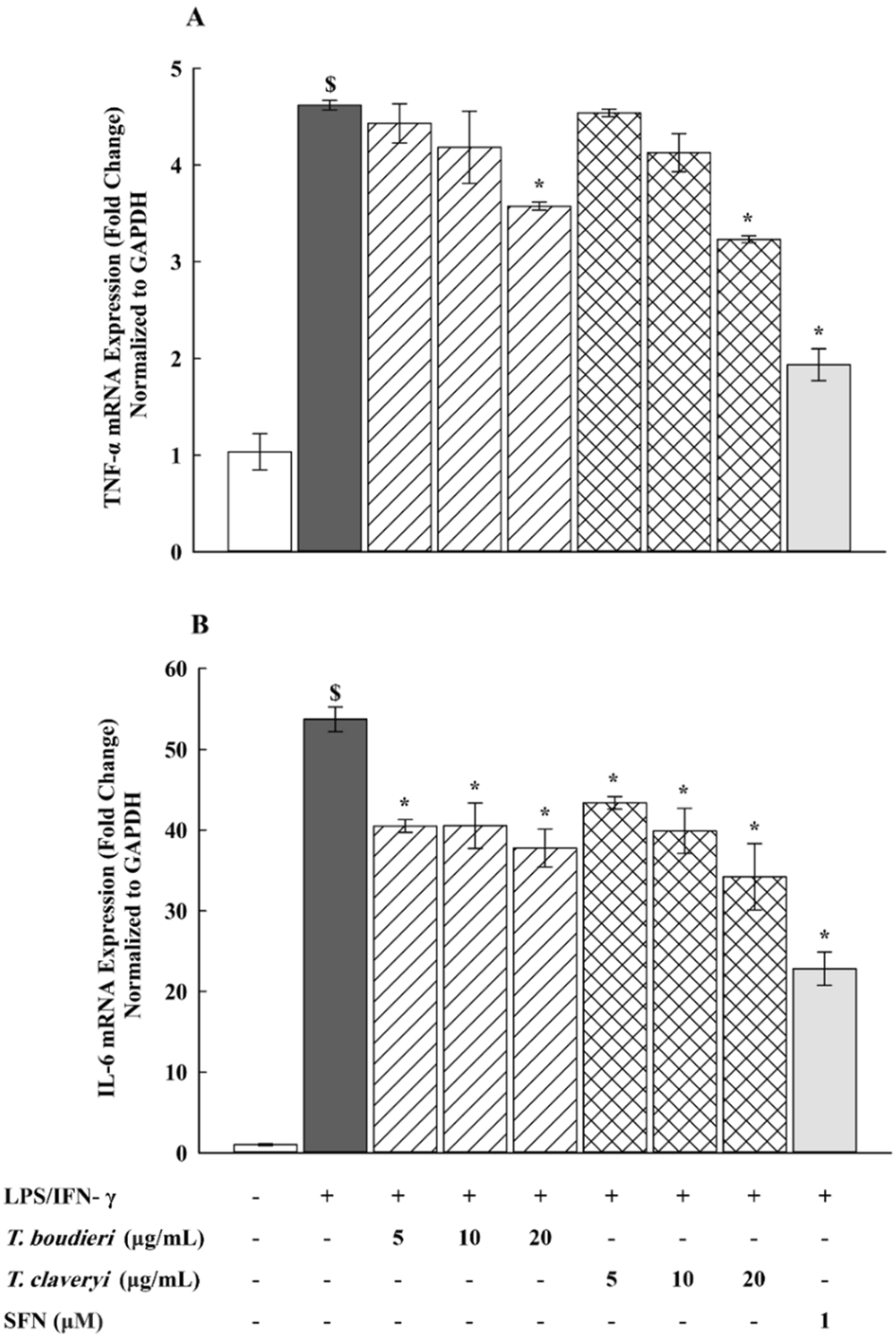
Effect of *T. boudieri* and *T. claveryi* extracts on the mRNA expression of TNF-α and IL-6 in LPS/IFN-γ-stimulated RAW 264.7 cells. RAW 264.7 cells were stimulated with LPS/IFN-γ (100 ng/10 U/mL) and co-incubated with *T. boudieri* and *T. claveryi* extracts at concentrations of 5, 10, and 20 µg/mL for 6 h. SFN (1 µM) was used as a positive control. TNF-α (**A**) and IL-6 (**B**) mRNA expression levels were analyzed by qPCR. Data are expressed as mean ± SEM (n = 3). Statistical significance was calculated by one-way ANOVA followed by Student–Newman–Keuls post-hoc test. $*P* < 0.05 versus control. *P < 0.05 versus LPS/IFN-γ.

To examine the potential anti-inflammatory effects of *Terfezia* extracts on the mRNA expression of pro-inflammatory cytokines TNF-α and IL-6, RAW 264.7 cells were treated with *T. boudieri* and *T. claveryi* extracts at concentrations of 5, 10, and 20 µg/mL in the presence of LPS/IFN-γ (100 ng/10 U/mL) for 6 h. SFN (1 µM) was used as a positive control. The mRNA expression of TNF-α and IL-6 was then determined using qPCR. Figure 5 shows that incubation with LPS/IFN-γ significantly increased the mRNA expression of TNF-α and IL-6 by 447 and 5278%, respectively, compared to the untreated controls. However, when the LPS/IFN-γ-stimulated cells were co-treated with *Terfezia* extracts, *T. boudieri* and *T. claveryi* at only 20 µg/mL significantly reduced the mRNA expression of TNF-α by 23, and 30%, respectively (Fig. 5A). Moreover, the mRNA expression of IL-6 was significantly downregulated by treatment with *T. boudieri* (5, 10, and 20 µg/mL) by 23, 25, and 30%, respectively, and also downregulated by treatment with *T. claveryi* (5, 10, and 20 µg/mL) by 20, 27, and 40%, respectively, as shown in Fig. 5B. Both extracts suppressed the LPS/IFN-γ-induced upregulation of TNF-α and IL-6 mRNA expression levels in a concentration-dependent manner. SFN (1 µM) also achieved a statistically significant inhibition of TNF-α and IL-6 by around 58%.

### Effect of *T. boudieri* and *T. claveryi* extracts on the mRNA expression of HO-1 and OSGIN1 in LPS/IFN-γ-stimulated RAW 264.7 cells

**Figure 6:**
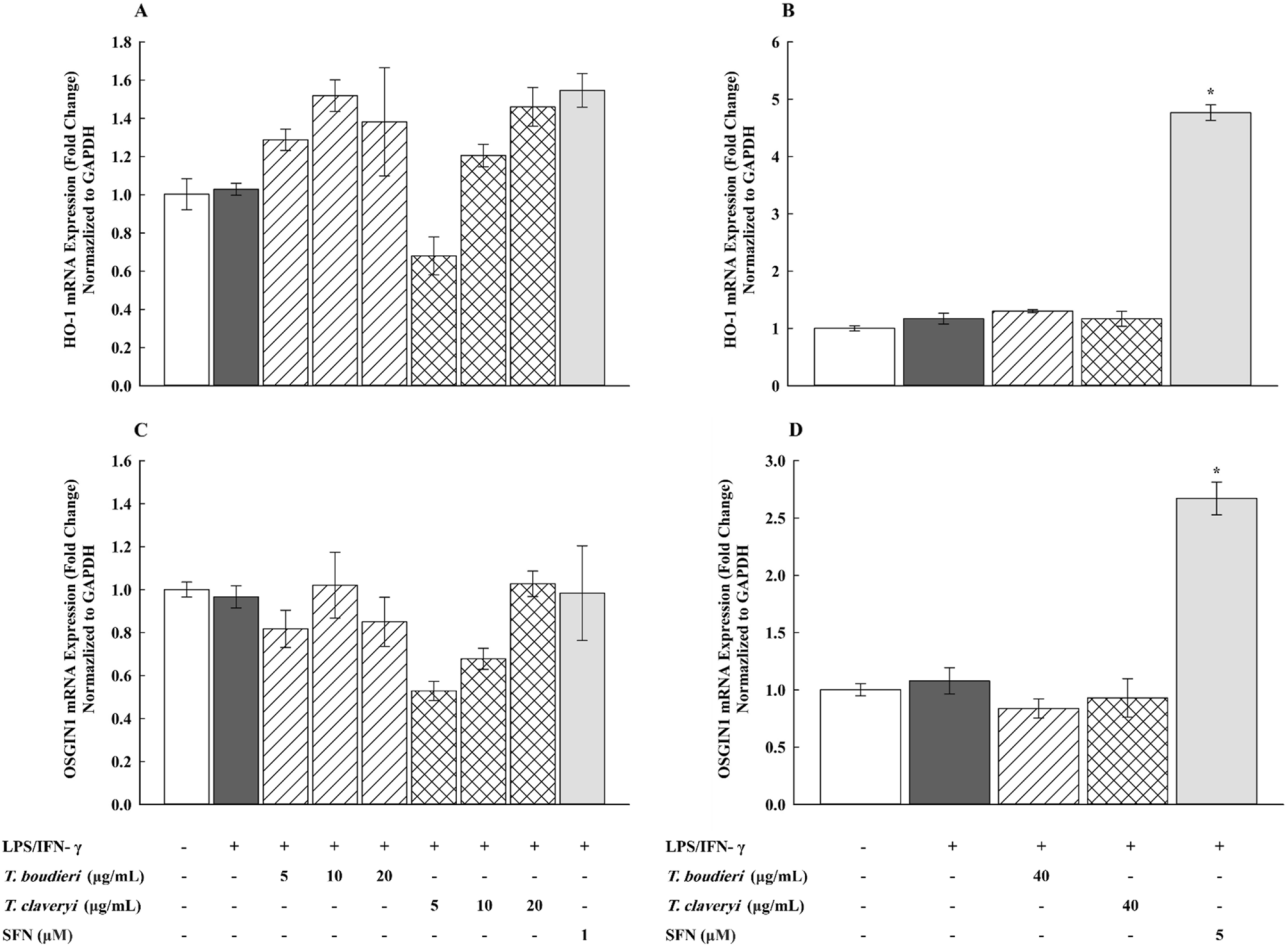
Effect of *T. boudieri* and *T. claveryi* extracts on the mRNA expression of HO-1 and OSGIN1 in LPS/IFN-γ-stimulated RAW 264.7 cells. RAW 264.7 cells were stimulated with LPS/IFN-γ (100 ng/10 U/mL) and co-incubated with *T. boudieri* and *T. claveryi* extracts at concentrations of 5, 10, 20, and 40 µg/mL for 6 h. SFN at concentrations of 1 and 5 µM was used as a positive control. The mRNA expression levels of HO-1 (**A**, **B**) and OSGIN1 (**C**, **D**) were analyzed by qPCR. Data are expressed as mean ± SEM (n = 3). Statistical significance was calculated by one-way ANOVA followed by Student–Newman–Keuls post-hoc test. $*P* < 0.05 versus control. *P < 0.05 versus LPS/IFN-γ.

To determine whether the anti-inflammatory effects of *Terfezia* extracts were related to modulation of the Nrf2 signaling pathway, RAW 264.7 cells were firstly treated with *T. boudieri* and *T. claveryi* extracts at concentrations of 5, 10, and 20 µg/mL in the presence of LPS/IFN-γ (100 ng/10 U/mL) for 6 h. SFN (1 µM) was used as a positive control. qPCR was then performed to measure the mRNA expression of Nrf2 targets (HO-1 and OSGIN1). The results depicted in Fig. 6A,C indicate all tested concentrations of *T. boudieri*, *T. claveryi,* and SFN did not lead to a significant activation of the mRNA expression of HO-1 and OSGIN1. Consequently, the concentration of *T. boudieri* and *T. claveryi* was increased to a non-cytotoxic concentration of 40 µg/mL. SFN was also increased up to 5 µM. As shown in Fig. 6B,D, SFN (5 µM) significantly upregulated the mRNA expression of HO-1 and OSGIN1 by 407 and 248%, respectively. Conversely, *T. boudieri* and *T. claveryi* (40 µg/mL) did not exhibit any significant effect on either HO-1 or OSGIN1 mRNA expression.

### Effect of *T. boudieri* and *T. claveryi* extracts on the miRNA expression of miR-21, miR-146a, and miR-155 in LPS/IFN-γ-stimulated RAW 264.7 cells

**Figure 7:**
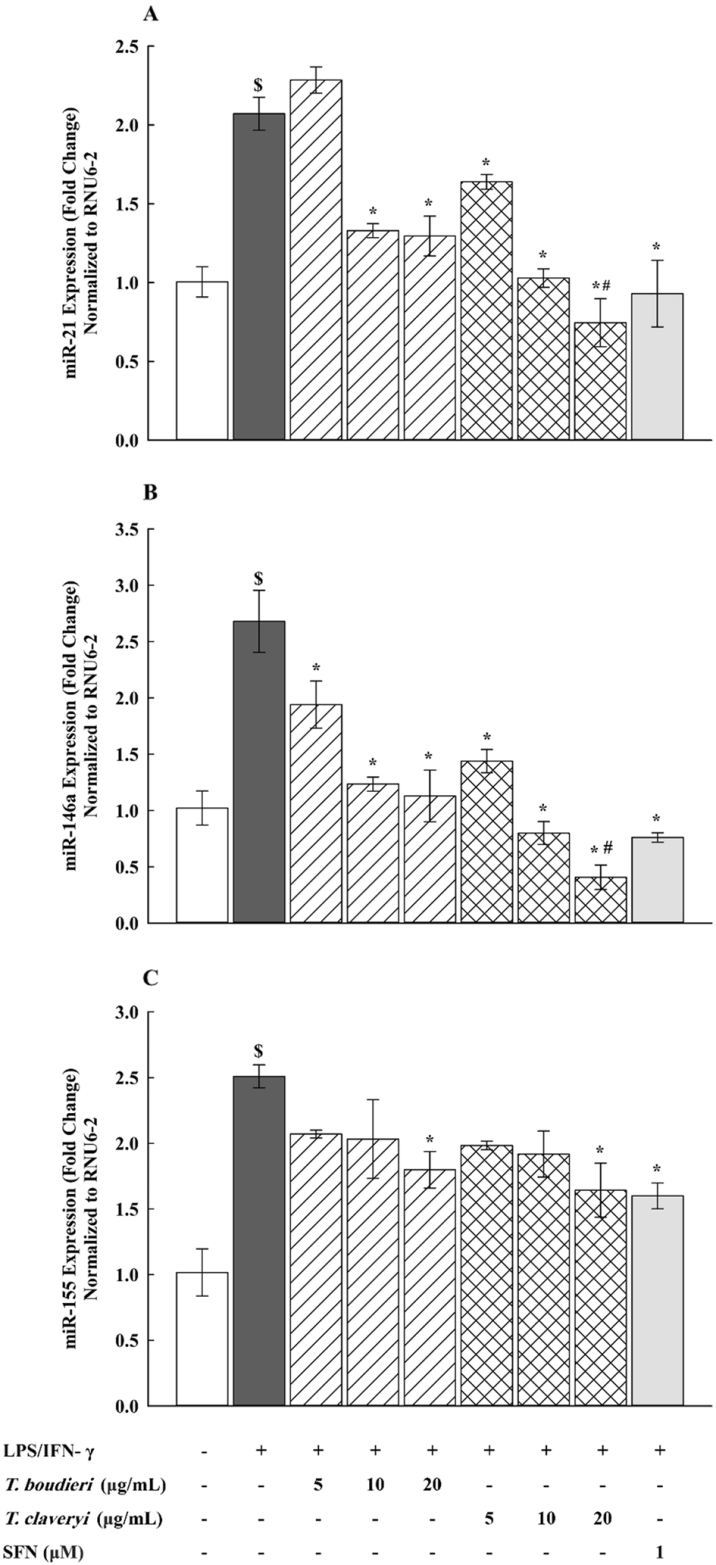
Effect of *T. boudieri* and *T. claveryi* extracts on the miRNA expression of miR-21, miR-146a, and miR-155 in LPS/IFN-γ-stimulated RAW 264.7 cells. RAW 264.7 cells were stimulated with LPS/IFN-γ (100 ng/10 U/mL) and co-incubated with *T. boudieri* and *T. claveryi* extracts at concentrations of 5, 10, and 20 µg/mL for 6 h. SFN (1 µM) was used as a positive control. The miRNA expression levels of miR-21 (**A**), miR-146a (**B**), and miR-155 (**C**) were analyzed by qPCR. Data are expressed as mean ± SEM (n = 3). Statistical significance was calculated by one-way ANOVA followed by Student–Newman–Keuls post-hoc test. $*P* < 0.05 versus control. *P < 0.05 versus LPS/IFN-γ. #*P* < 0.05, *T. boudieri* at any given concentration versus *T. claveryi* at the corresponding concentration.

To investigate whether the anti-inflammatory effects of *Terfezia* extracts were associated with epigenetic modulation of gene expression, RAW 264.7 cells were treated with *T. boudieri* and *T. claveryi* extracts at concentrations of 5, 10, and 20 µg/mL in the presence of LPS/IFN-γ (100 ng/10 U/mL) for 6 h. SFN (1 µM) was used as a positive control. qPCR was then performed to measure the miRNA expression of miR-21, miR-146a, and miR-155. As shown in Fig. 7**,** miR-21, miR-146a, and miR-155 were significantly upregulated by 206, 263, and 247%, respectively, in LPS/IFN-γ-induced cells compared to the untreated controls and significantly reduced by treatment with *Terfezia* extracts in a dose-dependent manner. Significant differences were also observed in the inhibitory effects of *Terfezia* extracts on the miR-21, and miR-146a, with *T. claveryi* (20 µg/mL) being statistically significant compared to *T. boudieri* (20 µg/mL). Figure 7A illustrates that *T. boudieri* (10 and 20 µg/mL) caused a significant reduction in miR-21 upregulation by approximately 35%, whereas *T. claveryi* (5, 10, and 20 µg/mL) showed significant inhibition of miR-21 upregulation by 21, 50, and 64%, respectively. Similarly, in Fig. 7B* T. boudieri* (5, 10, and 20 µg/mL) significantly reduced the upregulation of miR-146a by 28, 54, and 58%, respectively, while *T. claveryi* (5, 10, and 20 µg/mL) demonstrated highly significant inhibition of the upregulated miR-146a by 46, 70, and 85%, respectively. In Fig. 7C* T. boudieri* and *T. claveryi* at only 20 µg/mL significantly decreased miR-155 upregulation by 28 and 35%, respectively. Additionally, SFN (1 µM) led to downregulation of miR-21, miR-146a, and miR-155 expression by 55, 72, and 36%, respectively.

### Effect of *T. boudieri* and *T. claveryi* extracts on the secretion of TNF-α and IL-6 proteins in LPS/IFN-γ-stimulated RAW 264.7 cells

**Figure 8:**
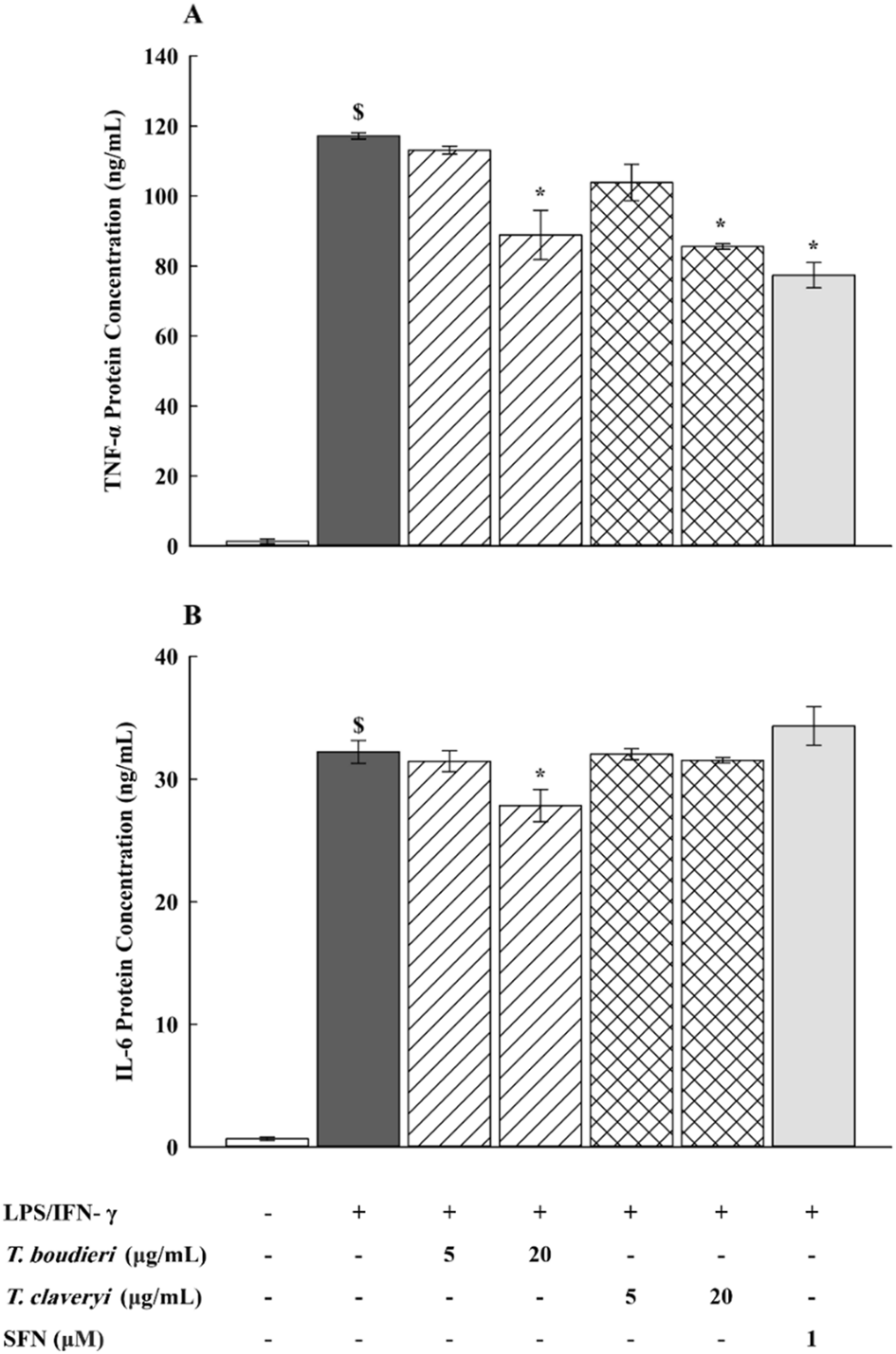
Effect of *T. boudieri* and *T. claveryi* extracts on the secretion of TNF-α and IL-6 proteins in LPS/IFN-γ-stimulated RAW 264.7 cells. RAW 264.7 cells were stimulated with LPS/IFN-γ (100 ng/10 U/mL) and co-incubated with *T. boudieri* and *T. claveryi* extracts at concentrations of 5 and 20 µg/mL for 24 h. SFN (1 µM) was used as a positive control. ELISA quantified TNF-α (**A**) and IL-6 (**B**) protein concentrations. Data are expressed as mean ± SEM (n = 3). Statistical significance was calculated by one-way ANOVA followed by Student–Newman–Keuls post-hoc test. $*P* < 0.05 versus control. *P < 0.05 versus LPS/IFN-γ.

To establish a relationship between the inhibitory effects of *Terfezia* on TNF-α and IL-6 mRNA expression and their corresponding secretion levels, RAW 264.7 cells were treated with *T. boudieri* and *T. claveryi* extracts at concentrations of 5 and 20 µg/mL in the presence of LPS/IFN-γ (100 ng/10 U/mL) for 24 h. SFN (1 µM) was used as a positive control. The concentration of TNF-α and IL-6 proteins secreted into the cell culture supernatant was measured using ELISA. As shown in Fig. 8, the production of TNF-α and IL-6 proteins was significantly increased in the cell culture medium of LPS/IFN-γ-induced cells compared to the untreated controls. However, when the cells were co-treated with *Terfezia* extracts, *T. boudieri* at 20 µg/mL significantly reduced TNF-α and IL-6 production by 24, and 14%, respectively. It was also found that *T. claveryi* at 20 µg/mL and SFN (1 µM) displayed a significant reduction in TNF-α production by 27 and 34%, respectively, but they did not exhibit any significant inhibition of IL-6 production (Fig. 8A,B).

### Effect of *T. boudieri* and *T. claveryi* extracts on the expression of iNOS and COX-2 proteins in LPS/IFN-γ-stimulated RAW 264.7 cells

**Figure 9:**
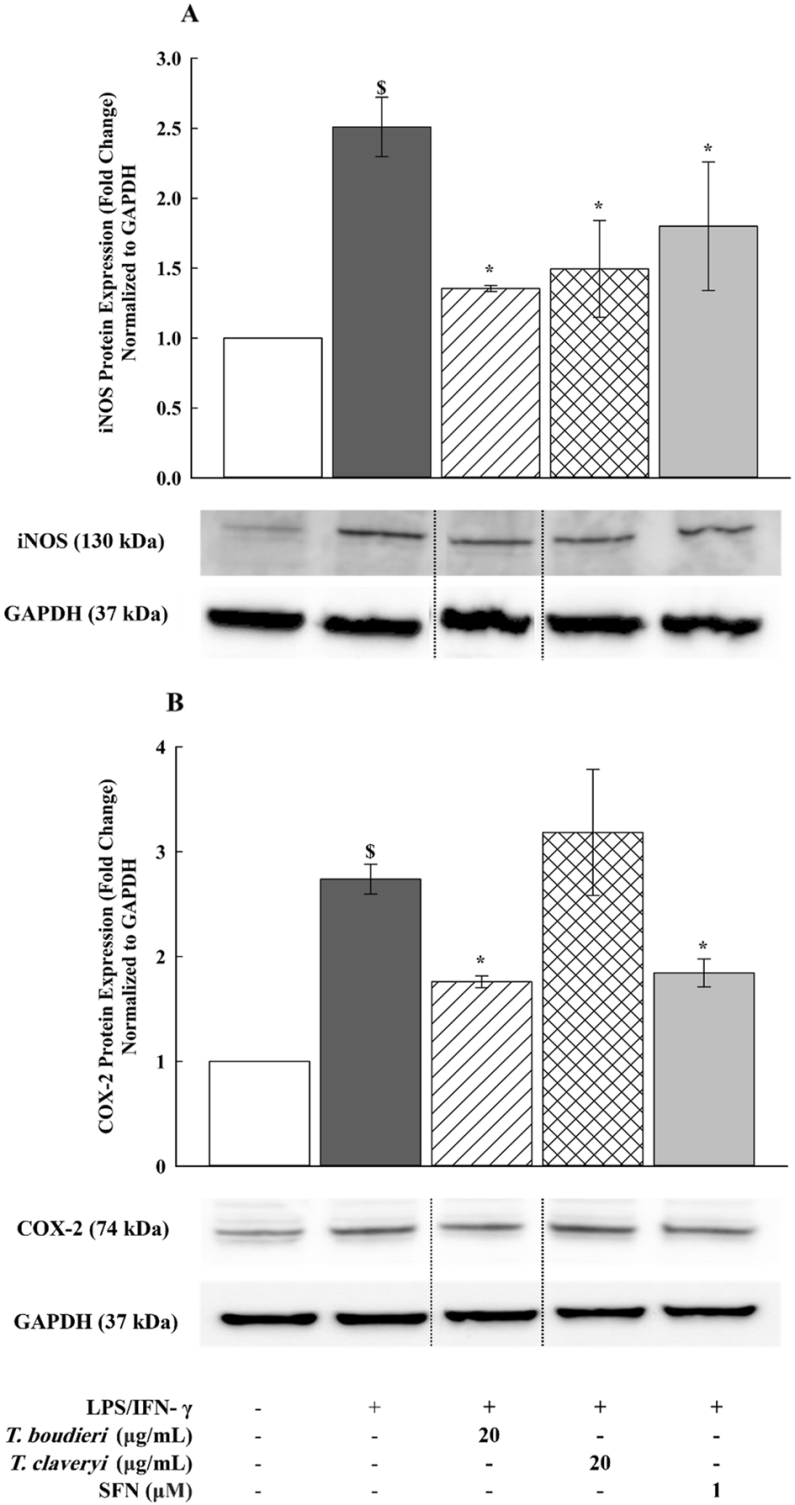
Effect of *T. boudieri* and *T. claveryi* extracts on the expression of iNOS and COX-2 proteins in LPS/IFN-γ-stimulated RAW 264.7 cells. RAW 264.7 cells were stimulated with LPS/IFN-γ (100 ng/10 U/mL) and co-incubated with *T. boudieri* and *T. claveryi* extracts at concentrations of 20 µg/mL for 24 h. SFN (1 µM) was used as a positive control. iNOS (**A**) and COX-2 (**B**) protein expression levels were determined by western blotting. GAPDH was used as a loading control. Samples were run on the same gel but were non-adjacent. Data are expressed as mean ± SEM (n = 3). Statistical significance was calculated by one-way ANOVA followed by Student–Newman–Keuls post-hoc test. $*P* < 0.05 versus control. *P < 0.05 versus LPS/IFN-γ.

To verify the inhibitory effect of *Terfezia* extracts on the iNOS and COX-2 expression at the protein level, RAW 264.7 cells were treated with *T. boudieri* and *T. claveryi* extracts at concentrations of 20 µg/mL in the presence of LPS/IFN-γ (100 ng/10 U/mL) for 24 h. SFN (1 µM) was used as a positive control. The expression of iNOS and COX-2 proteins were examined using western blotting. As shown in Fig. 9, LPS/IFN-γ significantly increased the expression of iNOS and COX-2 proteins by 251 and 274%, respectively, compared to the untreated controls. However, when the LPS/IFN-γ-stimulated cells were co-treated with *Terfezia* extracts, *T. boudieri* (20 µg/mL) significantly inhibited the iNOS and COX-2 expression by 46 and 36%, respectively. Similarly, *T. claveryi* (20 µg/mL) suppressed the expression of iNOS by 40%, but it did not demonstrate any significant effect on the COX-2 expression. SFN (1 µM) also inhibited the iNOS and COX-2 expression by 28 and 33%, respectively. The expression of the GAPDH, which was used as a loading control was not changed (Fig. 9A,B).

## Discussion

Inflammation is a highly regulated process that can be triggered by noxious stimuli, such as pathogens and toxins. Inflammation is, therefore, the first immunological line of defense through which the body can remove infection and repair tissue damage. The extent of the inflammatory response is critical because failure to eliminate the inflammatory trigger during acute inflammation leads to chronic inflammation, autoimmune reactions, and severe tissue damage. NSAIDs are the most used drugs in treating inflammation-associated diseases but have serious side effects^13^. Thus, multiple studies have been performed to explore alternative anti-inflammatory medicines of natural origin without the side effects of NSAIDs. Natural products are considered potential sources of novel anti-inflammatory agents, which can contribute to developing innovative therapeutics^39^. Desert truffles were reported to treat several inflammatory diseases^25^. Nevertheless, the mechanisms behind their anti-inflammatory activities in RAW 264.7 macrophages remain unclear. Hence, the anti-inflammatory properties of two major desert truffles, *T. boudieri* and *T. claveryi* have been examined in the present study.

Our metabolomic analysis of *T. boudieri* and *T. claveryi* revealed that their extracts contained several biologically active substances, including alkaloids, phenolics, amino acids, and fatty acids, which may be responsible for their anti-inflammatory activities. It has been reported from previous studies that natural alkaloids exhibited anti-inflammatory activities through various targets and cell signaling pathways^40,41^. Our results indicated the appearance of 4-Hydroxy-2,2,6,6-tetramethylpiperidine-1-oxyl (tempol) and piperlongumine alkaloids in the chemical profile of *T. claveryi*. In an in vitro study to evaluate the anti-inflammatory activities of tempol, it was determined that tempol treatment inhibited NO production in LPS-induced macrophages as well as suppressed the expression of cytokines (TNF-α, IL-1β, and IL-6) and inflammatory enzymes (iNOS, and COX-2) in IL-1β-stimulated chondrocytes^42^. Similarly, in activated macrophages, Sun et al. demonstrated that piperlongumine or its analog inhibited LPS-induced production of NO and PGE2 by downregulating the expression of iNOS and COX-2, respectively. They also reported that piperlongumine analog reduced LPS-dependent induction of NF-κB and MAPKs^43^. Phenolics are among the most essential classes of secondary metabolites, present in the fungal fruiting bodies with proven anti-inflammatory activities^44^. From our analysis, *T. boudieri* was characterized by the presence of phenolic compounds, particularly scopoletin, which exerted anti-inflammatory activities in croton oil-treated mouse ears by reducing the overproduction of TNF-α and PGE2^45^. The anti-inflammatory actions of truffles can be attributed to their amino acid contents, which are associated with prostaglandin metabolism^46^. Pyroglutamic acid was identified in *T. claveryi*, and its derivatives significantly inhibited the secretion of NO, TNF-α, and IL-6 in LPS-treated RAW 264.7 macrophages^47^. On the other hand, *T. boudieri* was rich in stachydrine, which exhibited anti-inflammatory properties by suppressing the levels of NO, PGE2, iNOS, COX-2, TNF-α, and IL-6 as well as blocking the IL-1β-mediated potentiation of NF-κB pathway in IL-1β-induced chondrocytes^48^. Furthermore, *T. boudieri* contained *N*-acyl homoserine lactone, and analogues of this compound have been shown to inhibit cytokine release of IL-6 and IL-8 in different in vitro-stimulated cell lines^49^. Fatty acids present in truffles potentially support their anti-inflammatory characteristics because of their high contents of polyunsaturated fatty acids (PUFAs), which are the precursors of the eicosanoids^46^. The metabolome of both *Terfezia* species was distinguished by the abundance of PUFAs, especially linoleic acid. Saiki et al. indicated that linoleic acid extracted from *Agaricus brasiliensis* reduced NO secretion and inhibited the expression of TNF-α, IL-6, IL-1β, and iNOS in RAW 264.7 macrophages. Linoleic acid also suppressed the activation of the NF-κB pathway^50^. The metabolite profiling of *T. boudieri* and *T. claveryi* contained phenylacetic acid and cinnamic acid, respectively, two active aromatic compounds with anti-inflammatory activities^51,52^.

SFN was used as a positive control in this study because it possesses a potent anti-inflammatory activity, mediated through suppressing the TLR4 oligomerization^53^. Additionally, Heiss et al. indicated that SFN reduced the production of the inflammatory mediators, including NO, PGE_2_, and TNF-α, accompanied by downregulation of iNOS and COX-2 proteins in LPS-induced RAW 264.7 cells^54^. SFN-mediated anti-inflammatory activity has been attributed in part to the activation of the Nrf2, as reported by Lin et al.^55^. In addition to the previous studies, Saleh et al. demonstrated that SFN modulated the TLR-associated miRNAs, such as miR-146a and miR-155, in LPS/IFN-γ-induced RAW 264.7 cells^56^. As a result, SFN can be considered as a suitable benchmark for assessing the anti-inflammatory properties of other medications.

Macrophages play pivotal roles during the inflammatory response, such as phagocytosis of microbes, antigen presentation, and secretion of inflammatory mediators. Moreover, macrophages are essential for maintaining homeostasis and tissue regeneration after injury^57^. Therefore, in vitro models of macrophages are important tools in evaluating the efficacy of the anti-inflammatory drugs through assessing the inflammatory response and cytotoxicity. RAW 264.7 macrophage cells are generally used to investigate the anti-inflammatory properties of drugs, and significantly activated by LPS and/or IFN-γ^58,59^. IFN-γ is included in combination with LPS in the macrophage polarization, and the induction of macrophages with either one of them, leads to the secretion of several inflammatory mediators^60^. In the current study, to examine the effects of *T. boudieri* and *T. claveryi* extracts on the inflammatory pathways, RAW264.7 cells were stimulated with LPS (100 ng/mL) plus IFN-γ (10 U/mL). The rationale for selecting LPS/IFN-γ instead of LPS alone was based on our optimization experiments. We found that RAW 264.7 cells treated with LPS alone exhibited weak immune responses, and the addition of IFN-γ was able to potentiate the immune response by activating signaling pathways that lead to the production of inflammatory cytokines and NO.

The increased production of NO, as measured by the Griess assay, supported this finding. Our results align with the previously published study by Saleh et al., which has shown that LPS/IFN-γ produces a more robust immune response than LPS alone^56^. Initially, we determined the non-cytotoxic concentrations of *T. boudieri,* and *T. claveryi* extracts with or without LPS/IFN-γ in RAW 264.7 cells using an MTT assay. Our data showed that treatment of RAW 264.7 cells with *T. boudieri* and *T. claveryi* extracts in the presence or absence of LPS/IFN-γ was not associated with a decrease in the cellular viability at concentrations between 5–20 μg/mL. Therefore, 5, 10, and 20 μg/mL concentrations were selected for both extracts in all further experiments.

The inflammatory response is mediated by a wide range of mediators forming complex regulatory networks that prevent further tissue damage and restore the normal physiology of the inflamed tissues^2^. Once LPS/IFN-γ activates macrophages, the production of inflammatory mediators, including cytokines (e.g., TNF-α and IL-6), eicosanoids (e.g., prostaglandins), and NO is increased^61,62^. NO is a key signaling molecule that is vital to the inflammatory response. NO is released as a cellular signaling molecule to increase the vasodilation in blood vessels by activating iNOS, which subsequently leads to an apparent increase in the blood flow and recruitment of leukocytes to the region of inflammation^7,63^. Therefore, to investigate the anti-inflammatory effects of *T. boudieri* and *T. claveryi* extracts, we analyzed the NO production and the iNOS expression at the mRNA and protein levels in LPS/IFN-γ-stimulated RAW 264.7 cells. Since NO is rapidly oxidized to nitrite, the nitrite level in the culture medium was measured as an indicator of the NO production^64^. Here, we found that *T. boudieri* and *T. claveryi* extracts inhibited NO production in a dose-dependent manner, accompanied by a simultaneous reduction in the iNOS mRNA expression. These data were consistent with our Western blotting analysis, in which both extracts exhibited significant inhibition of the expression of iNOS protein. Our results indicate that *Terfezia* extracts effectively improve inflammatory conditions by inhibiting NO's overproduction and the expression of iNOS in LPS/IFN-γ-stimulated RAW 264.7 cells.

Prostaglandins are eicosanoid-derived molecules that participate in modulating numerous physiological processes, particularly during immune responses. Prostaglandins are produced through arachidonic acid metabolism by cyclooxygenases, which exist in two isoforms: COX-1 and COX-2. COX-1 is produced constitutively in most cells, whereas COX-2 is induced in response to inflammatory stimuli^65^. As a consequence, a plethora of pharmacological agents has been developed to suppress COX-2 activity and thereby mitigate the inflammatory response. Thus, to examine the suppressive effect of *T. boudieri* and *T. claveryi* extracts, we measured the mRNA and protein expression of COX-2 in LPS/IFN-γ-stimulated RAW 264.7 cells. Our study findings demonstrated that both extracts effectively reduced the mRNA expression of COX-2. Notably, *T. boudieri* exhibited more potent inhibitory action on both mRNA and protein levels. Taken together, these data suggest that the anti-inflammatory properties of *Terfezia* extracts are associated with the suppression of COX-2 expression in LPS/IFN-γ-stimulated macrophages.

Cytokines are essential signaling proteins regulating the crosstalk between different cell types involved in the immune and inflammatory response^12^. Among cytokines, TNF-α is the primary mediator of inflammation with several effects, including stimulating other cytokine secretion, activating cell adhesion molecules, and promoting cell growth and proliferation^3,12^. IL-6 is another important cytokine released during inflammation, and its dysregulation causes a variety of inflammatory disorders^66^. Hence, the dysregulation in the production of inflammatory cytokines is commonly related to inflammatory diseases, making them potential therapeutic targets. In the current study, we assessed the expression of pro-inflammatory cytokines TNF-α and IL-6 at the mRNA and protein levels to identify the possible effects of *T. boudieri* and *T. claveryi* extracts on the inflammation mediated by LPS/IFN-γ in RAW 264.7 cells.

Interestingly, *T. boudieri* extract decreased the expression of TNF-α and IL-6 in a dose-dependent manner at the mRNA and protein levels. In contrast, *T. claveryi* was found to decrease the mRNA expression of TNF-α and IL-6, along with the inhibition of TNF-α protein production, while no significant effect was detected on IL-6 protein levels. Consistent with our results, Darwish et al. revealed that *T. claveryi* displayed anti-inflammatory activity by reducing TNF-α, IL-1β, and IFN-γ in LPS-stimulated WBCs^67^. These results reveal that *Terfezia* extracts possess anti-inflammatory properties via suppressing the pro-inflammatory cytokines involved in the inflammatory process.

Following the earlier findings, it can be observed that *T*. *claveryi* treatment decreased the mRNA expression of COX-2 and IL-6 but did not show the same effect on the protein levels. Several possibilities could explain this discrepancy, including differences in the time frame of our measurements, post-transcriptional modifications, protein stability, and other regulatory factors^68^. Firstly, the time frame of our measurements could be a contributing factor. We measured the mRNA expression levels at 6 h after stimulation with LPS/IFN-γ, while protein expression levels were measured at 24 h. Therefore, it is possible that *T. claveryi* treatment had a transient effect on the mRNA levels that were not reflected at the protein expression levels at 24 h. Secondly, post-transcriptional modifications can affect mRNA's stability and translation efficiency, which may not always be reflected at the protein expression levels. After transcription, mRNA undergoes several post-transcriptional modifications such as splicing, polyadenylation, and transport from the nucleus to the cytoplasm, affecting its stability and translation efficiency^69^. Thirdly, protein stability can also contribute to the discrepancy between mRNA and protein expression levels. Even if the mRNA levels are decreased, the protein levels may remain stable due to post-translational modifications or other factors^68^.

Nrf2 is a key transcription factor, playing a central role in the inflammation signaling pathways and oxidative stress responses. Therefore, the present study examined whether the anti-inflammatory effects of *T. boudieri* and *T. claveryi* extracts were related to activation of the Nrf2 signaling pathway in RAW 264.7 cells induced by LPS/IFN-γ through assessing the gene expression of Nrf2 target genes, HO-1 and OSGIN1. Inflammatory cells produce numerous inflammatory mediators, which subsequently attract more inflammatory cells to the site of injury, leading to increased oxidative stress levels. Meanwhile, persistent oxidative stress is associated with chronic inflammation. Nrf2 signaling pathway is also essential in reducing inflammation-related disorders, including atherosclerosis, asthma, autoimmune diseases, and rheumatoid arthritis^15^. Several studies have reported that the activation of the Nrf2 signaling pathway suppressed cytokines, chemokine, iNOS, and COX-2 secretion, which modulate the NF-kB and other inflammatory cascades that regulate the transcription and activity of downstream target proteins during the inflammation process^19^. Our preliminary results demonstrated that exposure to various concentrations (5, 10, and 20 µg/mL) of *T. boudieri*, *T. claveryi*, as well as SFN at a concentration of 1 µM, did not result in a significant upregulation of the mRNA expression of HO-1 and OSGIN1. Since SFN is known to be a potent activator of Nrf2, we initially expected it to upregulate the expression of Nrf2 target genes. However, we did not observe any significant effects on the expression of these genes at a concentration of 1 µM. Therefore, we increased the concentration of SFN to 5 µM to determine if this would lead to changes in Nrf2 target genes expression. The concentration of *T. boudieri* and *T. claveryi* was also increased to a non-cytotoxic concentration of 40 µg/mL, as assessed by MTT assay. As opposed to our initial results, we observed that SFN at a concentration of 5 µM activated the gene expression of HO-1 and OSGIN1 in our study. This is consistent with previous research by Doss et al., who reported a significant increase in HO-1 mRNA levels in erythroid cells treated with 5 µM SFN, whereas lower concentrations (100 nM-1 µM) did not produce the same effect^70^. On the other hand, treatment with *T. boudieri* and *T. claveryi* at concentration of 40 µg/mL did not show any significant induction on the gene expression of HO-1 and OSGIN. These results imply that the anti-inflammatory effects of *Terfezia* extracts are not mediated through Nrf2 activation.

In order to gain a deeper understanding of the anti-inflammatory mechanisms of *Terfezia* extracts, this study explored the effects of *T. boudieri* and *T. claveryi* extracts on the expression of inflammatory miRNAs, including miR-21, miR-146a, and miR-155 in RAW 264.7 cells activated with LPS/IFN-γ. The selection of these miRNAs in the study was based on their relevance to the TLR4 signaling pathway^71^. miRNAs play significant roles in regulating immune cell functions in both innate and adaptive immune systems through targeting inflammation-related pathways, such as TLR4^72^. miR-21 is a negative regulator of the TLR4-induced immune response that suppresses the NF-κB activity and promotes the production of the anti-inflammatory cytokine IL-10 by targeting the programmed cell death protein 4 (PDCD4)^73^. Furthermore, miR-146a was identified to be an NF-κB-dependent gene that targets interleukin-1 receptor-associated kinases1 (IRAK1) and TNF receptor-associated factor 6 (TRAF6) in the TLR4 signaling pathway, suggesting it as a negative regulator of the innate immune response^74^. Moreover, miR-155 is known to play a significant role in inflammation by targeting multiple proteins involved in the TLR4 signaling pathway^75^. Ceppi et al. reported that miR-155 negatively regulated the inflammatory response to LPS in monocyte-derived dendritic cells. This negative regulation by miR-155 was related to its ability to target TAB2, suppressing its activation of TAK1, and thus the NF-κB and MAPK^76^. The results of the present study demonstrated upregulation of miR-21, miR-146a, and miR-155 in response to LPS/IFN-γ stimulation, consistent with previous studies^77–79^. Interestingly, treatment with *T. boudieri* and *T. claveryi* extracts was observed to inhibit the LPS/IFN-γ-induced upregulation of these miRNAs in a concentration-dependent manner, suggesting that both *Terfezia* extracts suppress the inflammatory response in activated macrophages through regulating the expression of miR-21, miR-146a, and miR-155.

Nrf2 activation is known to regulate inflammation by activating miRNAs, such as miR-21, miR-146a, and miR-155^80^. However, our results demonstrate that the anti-inflammatory properties of *T*. *boudieri* and *T. claveryi* are independent of Nrf2 activation. As such, the effects observed at the miR-21, miR-146a, and miR-155 are equally independent of any effects at the Nrf2 level. Although the specific mechanisms involved are not yet fully understood, our results suggest that these compounds may utilize alternative pathways to regulate inflammation. One possible mechanism is the inhibition of NF-κB signaling, a central inflammation regulator. While some natural compounds have been shown to inhibit NF-κB signaling, we did not investigate this pathway in our study.

Nevertheless, our findings are supported by similar results regarding the effects of some biologically active substances in *T. boudieri* and *T. claveryi* metabolome suggesting that NF-κB signaling may indeed play a role in the observed effects^43,48,50^. In addition, previous research from our laboratory has also suggested a role for Glycogen synthase kinase 3 (GSK3) in mediating anti-inflammatory response that is also Nrf2 independent^81^. As such, further research is necessary to determine the involvement of these pathways in the anti-inflammatory effects of *T. boudieri* and *T*. *claveryi*. Overall, our study suggests that these compounds may have the potential as alternative anti-inflammatory agents that act through non-traditional pathways.

## Conclusion and future perspectives

In conclusion, our results suggest that targeting specific inflammatory mediators associated with TLR4-mediated signaling could be an effective therapeutic approach for mitigating inflammation induced by LPS/IFN-γ in RAW 264.7 macrophages. The current study highlights the potential anti-inflammatory properties of *T. boudieri* and *T. claveryi* extracts in regulating the inflammatory response in LPS/IFN-γ-stimulated RAW 264.7 macrophages through modulation of the TLR4 activation. The results of our experiments showed that treatment with these extracts led to a concentration-dependent reduction in the production of NO that coincided with the downregulation of iNOS at both the mRNA and protein levels. Both extracts downregulated the COX-2 mRNA expression, with *T. boudieri* further reducing the expression of COX-2 protein. Furthermore, *T. boudieri* extract dose-dependently downregulated TNF-α and IL-6 mRNA and protein levels, while *T. claveryi* extract showed significant inhibition of TNF-α and IL-6 mRNA expression without affecting IL-6 and COX-2 protein levels. This study also offers novel insights into the epigenetic suppressive properties of *T. boudieri,* and *T. claveryi* extracts on miR-21, miR-146a, and miR-155 expression in LPS/IFN-γ-induced RAW 264.7 macrophages, proposing that *Terfezia* extracts could exert anti-inflammatory activities by suppressing the miRNAs involved in the TLR4 signaling pathway. Additionally, our findings showed that *T. boudieri* and *T. claveryi* exhibited anti-inflammatory effects through an Nrf2-independent manner. Furthermore, our study demonstrated differences in the chemical composition and anti-inflammatory effects of *T. boudieri* and *T. claveryi* extracts, despite both species being desert truffles. These differences can be attributed to the distinct chemical compositions of the two species. These differences highlight the importance of identifying and characterizing the specific bioactive compounds in each species, which could have different pharmacological properties and therapeutic potential. Finally, the present study suggests that *T. boudieri* and *T. claveryi* could serve as promising alternative agents for treating inflammation. However, the efficacy and safety of these extracts in vivo should be evaluated using appropriate animal models in future studies.

## Supplementary Information

Supplementary Information.

## Data Availability

All data generated or analyzed during this study are included in this published article.

## Abbreviations

COX-2: Cyclooxygenase-2
ELISA: Enzyme-linked immunosorbent assay
HO-1: Heme oxygenase 1
IFN- γ: Interferon-gamma
IL-6: Interleukin-6
iNOS: Inducible nitric oxide synthase
LPS: Lipopolysaccharide
miRNA: MicroRNA
NF-κB: Nuclear factor-kappa B
NO: Nitric oxide
Nrf2: Nuclear factor erythroid 2–related factor 2
NSAIDs: Non-steroidal anti-inflammatory drugs
OSGIN1: Oxidative stress induced growth inhibitor 1
PGE2: Prostaglandin E2
PUFAs: Polyunsaturated fatty acids
SFN: Sulforaphane
TLR 4: Toll-like receptor 4
TNF-α: Tumor necrosis factor alpha
