# Supplementary material for: Terfezia boudieri and Terfezia claveryi inhibit the LPS/IFN-γ-mediated inflammation in RAW 264.7 macrophages through an Nrf2-independent mechanism
Source: Sci Rep. 2023 Jun 21;13:10106. doi: 10.1038/s41598-023-35612-8 (PMC10284807; doi:10.1038/s41598-023-35612-8)
Supplement: Supplementary file 1 — Supplementary Information. [file 41598_2023_35612_MOESM1_ESM.docx]

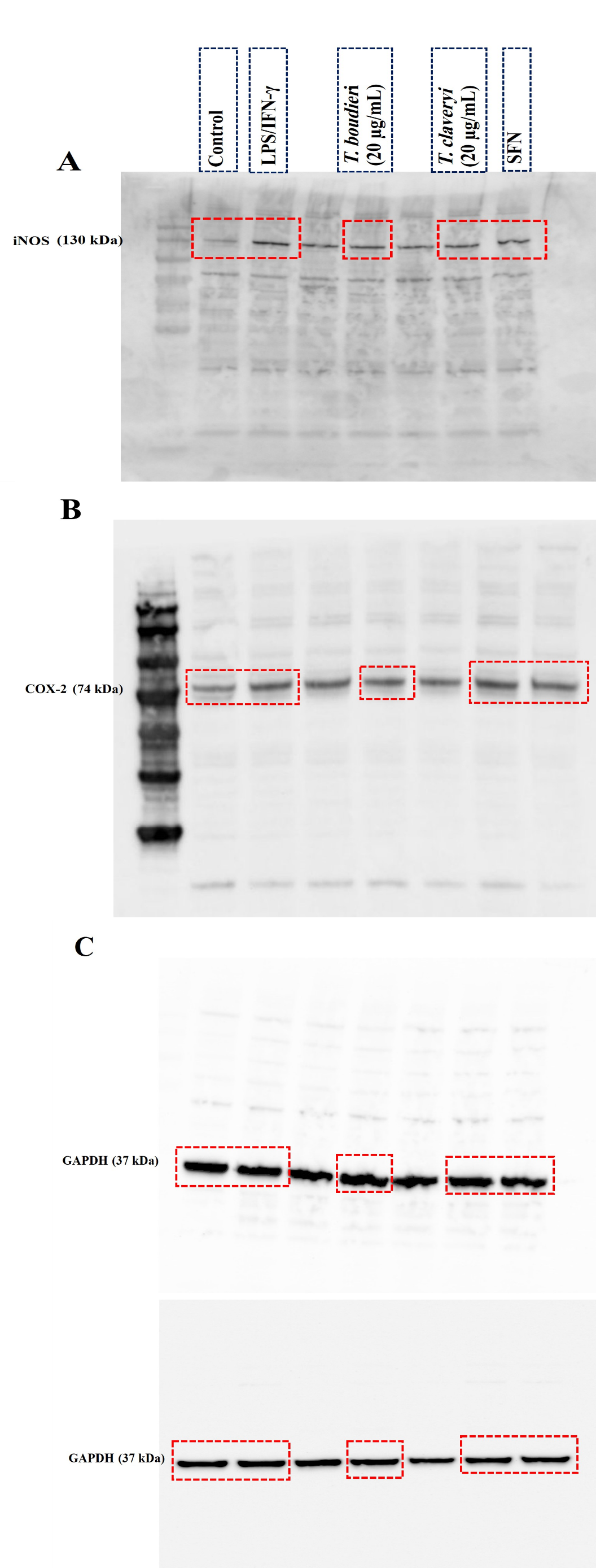


Supplementary Figure 1. Full uncropped Western blots shown in Figure 9A,B are presented in this figure. Cropped areas are highlighted by red box. RAW 264.7 cells were stimulated with LPS/IFN-γ (100 ng/10 U/mL) and co-incubated with *T. boudieri* and *T. claveryi* extracts at concentrations of 20 µg/mL for 24 h. SFN (1 µM) was used as a positive control. Protein expression levels of iNOS (A), COX-2 (B), and GAPDH (C) were quantified using western blotting. Samples were run on the same gel but were non-adjacent. Data are expressed as mean ± SEM (n = 3). Statistical significance was calculated by one-way ANOVA followed by Student–Newman–Keuls post-hoc test. $ *P* < 0.05 vs. control. * *P* < 0.05 vs. LPS/IFN-γ.
